# Long non-coding RNA SLC25A21-AS1 inhibits the development of epithelial ovarian cancer by specifically inducing PTBP3 degradation

**DOI:** 10.1186/s40364-022-00432-x

**Published:** 2023-01-30

**Authors:** Sihui Li, Shizhen Shen, Wanzhong Ge, Yixuan Cen, Songfa Zhang, Xiaodong Cheng, Xinyu Wang, Xing Xie, Weiguo Lu

**Affiliations:** 1 Women’s Reproductive Health Laboratory of Zhejiang Province; Women’s Hospital; School of Medicine, Zhejiang University, Hangzhou, 310006 China; 2 Department of Gynecologic Oncology; Women’s Hospital, School of Medicine, Zhejiang University, Hangzhou, 310006 China; 3 Cancer Center, Zhejiang University, Hangzhou, 310058 China; 4 Division of Human Reproduction and Developmental Genetics, Zhejiang Provincial Key Laboratory of Precision Diagnosis and Therapy for Major Gynecological Diseases, Women’s Hospital, Zhejiang University School of Medicine, Hangzhou, 310006 China; 5 Institute of Genetics, Zhejiang University, Hangzhou, 310058 China

**Keywords:** SLC25A21-AS1, PTBP3, ubiquitination, epithelial ovarian cancer, long noncoding RNAs

## Abstract

**Background:**

Epithelial ovarian cancer (EOC) is a highly prevalent disease that rapidly metastasizes and has poor prognosis. Most women are in the middle or late stages when diagnosed and have low survival rates. Recently, long non-coding RNAs (lncRNAs) were recognized to play pivotal roles in the development of EOC.

**Methods:**

The expression of SLC25A21 antisense RNA 1 (SLC25A21-AS1) and Polypyrimidine Tract Binding Protein 3 (PTBP3) in EOC cells was assessed via qPCR. The proliferation activity of these cells was detected by EdU and Cell counting kit-8 (CCK8) assays, while the death rate of apoptotic cells and the cell cycle were detected by flow cytometry. Detection of cell transfer rate by transwell assay. Protein expression was measured through western blotting. Interactions between SLC25A21-AS1 and PTBP3 were detected through RNA immunoprecipitation (RIP), IF-FISH co-localization experiments and electrophoretic mobility shift assay (EMSA). The *in vivo* importance of SLC25A21-AS1 as a tumor suppressor modulator was assessed using murine xenograft models.

**Results:**

The lncRNA SLC25A21-AS1 has negligible expression in ovarian cancer tissues compared with that in normal ovarian tissues. A series of functional experiments revealed that the upregulation of SLC25A21-AS1 markedly blocked the proliferation and metastasis of EOC cells *in vitro*, while its downregulation had the opposite effect. Overexpression of SLC25A21-AS1 in a nude mouse model of EOC *in vivo* resulted in slower tumor growth and weakened metastatic potential. Moreover, SLC25A21-AS1 reduced the protein stability of PTBP3 and promoted its degradation. A series of subsequent experiments found that SLC25A21-AS1 inhibits EOC cell proliferation and metastasis by modulating PTBP3 through the ubiquitin-proteasome pathway and that the combination of SLC25A21-AS1 and PTBP3 provides the necessary conditions for the for the function to be realized.

**Conclusions:**

Our research reveals the effect of SLC25A21-AS1 in EOC development and suggests SLC25A21-AS1 can serve as a prognostic target by promoting the degradation of PTBP3 to improve patient survival.

**Supplementary Information:**

The online version contains supplementary material available at 10.1186/s40364-022-00432-x.

## Introduction

According to GLOBOCAN 2020 Global Cancer Statistics, the incidence of ovarian cancer (OC) ranked the third in gynecological tumors and the second in female reproductive system malignancy. Among gynecological malignant diseases, the mortality rate of ovarian cancer is second only to cervical cancer in western countries. More than 310,000 new cases are diagnosed each year, with 200,000 women dying from OC. Due to the immaturity of clinical early diagnostic methods, 70% of patients are already in advanced stages when they are first diagnosed with EOC [1, 2]. The most common type of OC is EOC. Based on morphological characteristics, EOC is further divided into four subtypes: serous ovarian cancer, endometrioid ovarian cancer, clear cell type, and mucinous ovarian cancer. Among these, serous cancers are the most prevalent [3]. Therefore, it is important to understand the organizational structure of EOC [4, 5], its occurrence and development [6], and its drug resistance mechanisms [7]. Moreover, it is important to seek suitable biological targets [8] and treatments [9, 10] for this cancer. These aspects have been extensively investigated over the last few decades. Unfortunately, a lack of deep understanding in these exact areas has hampered the prevention and treatment of OC.

Findings from the Human Genome Project (HGP) have revealed that only a small portion of the genome encodes proteins [11]. The Encyclopedia of DNA Elements (ENCODE) project lists over 20 000 long non-coding RNAs (lncRNAs) that constitute the most functionally diverse class of non-coding transcripts [12]. The notion that lncRNAs have regulatory functions at multiple levels, such as a role in genome dynamics, embryogenesis and differentiation, gene expression, and even protein stability, has become evident [13, 14]. Studies have reported that lncRNAs carry abnormal gene mutations and their aberrant expression in cancer can deregulate cell cycle and proliferation [15–17]; their association with OC metastasis has also been described that can be embedded in EVs secreted by ovarian cancer and mediate the MTT process through delivering it to mesothelial cells [18].. LncRNAs can undergo genome editing, regulate protein degradation and their loss of function inhibits interactions with these components [19]. Identification and directional regulation of different lncRNAs plays an important role in future clinical applications. Therefore, it is necessary to study the role of lncRNAs in OC to identify potential treatments.

Polypyrimidine tract-binding protein 3 (PTBP3) is a member of the heterogeneous nuclear ribonucleoproteins (hnRNP) family, and is homologous to PTBP1, which binds to both introns and exons, and likely mediates the regulation of alternative splicing [20]. Moreover, PTBP3 (ROD1) is an important RNA-binding protein. It regulates DNA damage through p53 signaling by binding to lncRNA Meg3 and protects endothelial function [21]. In tumors, PTBP3 often cooperates with lncRNAs to regulate signaling pathways, thereby affecting tumorigenesis and development. For example, PTBP3 promotes breast cancer development through lncRNA BCRT1 by interacting with microRNA (miR)-1303 [22]. However, the role of PTBP3 in EOC has not been commonly reported and remains largely unknown.

In this study, we identified a lncRNA (SLC25A21-AS1) on human chromosome 14 and investigated its function and mechanism of action in EOC. Our findings provide novel evidence that SLC25A21-AS1 regulates the proliferation and metastatic abilities of EOC cells; here, to the best of our knowledge, for the first time, we propose that SLC25A21-AS1 may be a potential target to inhibit the development of EOC.

## Materials and methods

### Analysis of DEGs in the lncRNA datasets of EOC

The GSE14407 [23] and GSE135886 [24, 25] datasets were downloaded from the GEO database. The GSE14407 dataset contained transcriptome microarray data of 12 normal ovary and 12 ovarian cancer samples, and GSE135886 dataset contained those of 6 normal ovary and 6 ovarian cancer samples. Setting FDR <0.05 and [log2 (fold change)]> 1 as the cutoff, differentially expressed genes (DEGs) were identified by package Limma using R software (version3.6.2). Genes that were co-up or down-regulated in these two datasets were obtained by Venn diagram analysis.

### Cell Culture

The cell culture protocols for the human epithelial serous EOC lines SKOV3(RRID:CVCL_0532) and 3AO have been described previously [7]. The human normal ovarian epithelial cell line IOSE-80 (RRID:CVCL_5546) was kindly donated by Prof. Lu of Zhejiang University. Growth of IOSE-80 cells was supported by Dulbecco's Modified Eagle Medium (DMEM), while the SKOV3 and 3AO cells were cultured in RPMI1640 medium (BasalMedia, China). The SKOV3-luc cells were well established with the peptase-expressing lentiviral construct and cultured in RPMI1640 medium. Cells were supplied with 10% fetal bovine serum for nutrition (Everyday Green, China) and incubated at 37 °C with 5% carbon dioxide.

### Immunofluorescence assay

The SKOV3 and 3AO cells (2 × 104) were prepared in 4-well petri dishes (Cellvis, USA). Four percent paraformaldehyde was added for 10 min after the cells were fully attached. Next, cells were treated with 2% bovine serum albumin (BSA) and incubated with the primary antibodies overnight; this process is optimally performed at 4 °C. After incubation, the secondary antibody was added; to ensure optimal effect, it is recommended to incubate at room temperature for over one hour, followed by staining with DAPI. Finally, the stained cells were observed with a confocal microscope and images were captured.

### Fluorescence in situ hybridization (FISH)

The cells were evenly seeded onto 4-well confocal-specific Petri dishes; efforts were made to avoid too much seeding so as not to affect observation. Cells were fixed with 4% paraformaldehyde for 10 min and washed with PBS. The pre-cooled permeabilization solution was added, and cells were stored at 4 °C for 5 min. We then added 200 μl of pre-hybridization solution for 30 min, followed by 2.5 μl of 20 μM lncRNA FISH Probe Mix or 100 μl of hybridization solution under dark conditions, and incubated overnight. After incubation, cells were washed with hybridization wash solution and PBS. Observation was under a confocal microscope and pictures were acquired. FISH assay was carried out using the Ribo™ Fluorescence In Situ Hybridization Kit (RiBio, China) as previously described [26]. The SLC25A21-AS1, 18S, and U6 probes were synthesized by RiBio.

### Lentiviruses, small interfering RNAs (siRNAs), and plasmids

Inoculating cells at 50–60% density in the well plate and completing the transfection within 18–24 h after inoculation can ensure better transfection efficiency. Transfection status was visualized using fluorescence microscopy within 48 h after inoculation; transfection efficiency was also determined by subsequent qRT-PCR. The luciferase-expressing SLC25A21-AS1 and PTBP3 lentiviral constructs (Ubi-MCS-firefly_ Luciferase-IRES-puromycin) were compounded by Genechem (China) and transfected in EOC cells following the manufacturer’s instructions. The human SLC25A21-AS1 overexpression plasmid, SLC25A21-AS1 truncated (T1–T5), and PTBP3 overexpression plasmid was synthesized by GenePharma (China). The X-treme GENE™ HP DNA Transfection Reagent (Roche, China) was utilized for plasmid diversion. Plasmid and siRNAs of SLC25A21-AS1 and PTBP3 were provided by Tsingke (China), including negative controls for both. The siRNA transfections were performed using the DharmaFECT transfection reagent (Thermo, USA). The constructs were identified by DNA sequencing.

### Cell counting kit-8 (CCK8) and EdU assays

CCK8 assays, after complete adherence, were performed according to the manufacturer’s instructions. Results were included for 96 h, with tests performed every 24 h. Reaction times remained the same for each time interval. It was recommended that 2–3 hours would be appropriate, and incubation should be performed in the dark. Absorbance was measured spectrophotometrically at 450 nm. The EdU Cell Proliferation Detection Kit from RiBio was used for EdU detection. Here, 105 cells/well were inoculated into 6-well plates, and EdU was added and fixed for 2 h at 37 °C. Incubation was with 100 μl of penetrant for 10 min, followed by 1x Apollo staining reaction solution and incubation in dark. After infiltration, DNA staining was performed. Detection was performed using immunofluorescence microscopy.

### Migration and invasion assays

For the migration experiments, treated SKOV3 and 3AO cells (1 × 10 [5] cells/well) were seeded in the upper chambers of 24-well plates (pore size, 8.0 μm; BD, USA). The medium in the chamber was serum-free, whereas the medium in the lower chambers contained 10% fetal bovine serum. SKOV3 cells were incubated for 6 h and 3AO cells for 12 h. Five fields of view were randomly selected to obtain images, and the average number of SKOV3 or 3AO cells in each field was calculated. In the invasion experiment, BD gel was placed in upper chambers before introducing cells for 30 min to solidify. Subsequent steps were identical to those for the migration experiments. The SKOV3 cells were removed after 8 h and the 3AO cells after 24 h for fixation and staining.

### Cell cycle assay and apoptosis assay

Tracking of cell cycle was accomplished using a Cell Cycle Staining Kit (MULTI SCIENCES, China). One milliliter of DNA Staining Solution and 10 μl permeabilization solution were added to EOC cells (1 × 10 [6]) and vortexed to mix before incubation at room temperature for 30 min in the dark. The lowest sample loading speed was selected for detection of cell cycle progression, which was performed using a flow cytometer. Apoptosis was detected using the Annexin V-FITC/PI apoptosis kit (MULTI SCIENCES, China). Cells (1 × 10 [6]) were pretreated with 500 μl of pre-chilled 1x Binding Buffer, resuspended in 5 μl Annexin V-FITC and 10 μl PI working solution and incubated in dark. Detection was performed using a flow cytometer.

### RNA-pull-down assay and protein mass spectroscopy (MS)

RNA pull-down assays were performed using a Pierce™ Magnetic RNA-Protein Pull-Down Kit (Thermo Scientific, Waltham, MA, USA). First, cells were lysed using the Thermo Scientific Pierce Ip Lysis Buffer, ensuring that protein concentration after cell lysis was > 2 mg/ml. Biotinylated probes were ordered from Tsingke (China) and labeled. A suspension of 50 ml of magnetic beads was washed with 20 mM Tris, followed by addition of an equal volume of 1x RNA Capture Buffer and 100 pmol of probe and incubation. An RNA-Protein binding system (according to the manufacturer’s instructions) was added, followed by overnight incubation. Magnetic beads were collected and subsequent WB studies were completed.

Technical support for protein MS analysis was provided by OE Biotech (Shanghai, China).

### RNA extraction and qRT-PCR analysis

Cellular RNA extraction utilized an RNA extraction kit (China). Collected cells (1 × 10 [6]) were treated with 500 μl of Lysis Buffer. Equal volume of absolute ethanol was added to the lysate, followed by centrifugation at 4 000 × g for 1 min. Five hundred microliters of Wash Buffer were added to the RNA column, centrifuged, and the lid was removed to dry the sample. Then, 20 μl of Elution Buffer was added onto the matrix and the sample was centrifuged at 12 000 × g for 1 min to obtain the RNA. Cytoplasmic and nuclear RNAs were isolated utilizing the PARIS kit (American Life Technologies) according to the manufacturer’s protocol. The PrimeScript RT kit (Takara, Japan) was utilized for RNA reverse transcription. The processes were carried out strictly in accordance with the manufacturer’s instructions. Furthermore, qRT-PCR analysis was conducted using the TB Green Master Mix Ex Taq (Takara, Japan) and a 7900HT Fast Real-Time PCR System (Life Technologies, USA). Relative mRNA and lncRNA expression levels were normalized to GAPDH using the 2^-ΔΔCt^ method.

### Western blot (WB)

WB assays were performed on 10% SDS-PAGE gels (GenScript, USA). After adding the extracted protein to the gel, protein separation was completed at 120–130 V. The process was performed on the eBlot L1 Protein Transfer System (GenScript). The blocked membrane was incubated with the primary antibody at 4 °C overnight. The secondary antibody was added after thorough washing, followed by incubation at room temperature for 1 h on a shaker. After washing the secondary antibody, the ImageQuant LAS 4000 mini (ImageQuant LAS 4000 mini, USA) was used for image acquisition.

### RNA immunoprecipitation (RIP) assay

A Magna RIP RNA-Binding Protein Immunoprecipitation Kit (Merck Millipore, Germany) was employed for the RIP assay. Briefly, cells (2 × 10 [7]) were harvested and resuspended in complete lysis buffer and then lysed to completion at −80 °C after 5 min of incubation on ice. A solution of 50 μl of magnetic beads was washed thoroughly. After adding 5 μg of the target antibody, incubation followed at room temperature for 30 min. Next, 0.5 ml of RIP wash buffer were added and vortexed briefly. Following this, the devotion RNA-binding protein-RNA complex was co-immunoprecipitated. The RIP immunoprecipitation buffer, prepared according to the manufacturer’s instructions, was added to the RIP lysate and immunoprecipitation buffer overnight with rotation to allow complete binding. Finally, the protein was fully digested and the RNA was purified. The salt solutions, precipitation enhancer and absolute ethanol were used for -80 °C storage for RNA precipitation. The next day, after centrifugation and washing, the pellets were naturally air-dried, and the obtained RNAs were dissolved in DEPC-treated water and placed on ice for use. The quality and abundance of SLC25A21-AS1, or its truncated variant (T1–T5), were detected using qRT-PCR.

### Electrophoretic mobility shift assay (EMSA)

Cells (1x10 [7]) were added to a lysis buffer containing a protease inhibitor and DTT. The samples were vortexed, lysed on ice for 30 min, and centrifuged. An RNA-EMSA reagent was added to avoid air bubbles and left at room temperature. This operation can effectively avoid non-specific binding between the protein and probe. The labeled probe was mixed in and co-cultivated for 20 min, and the samples were used for electrophoresis. Detection was performed using an RNA EMSA kit (Axl-bio, China).

### Xenograft tumorigenesis

Twenty-one nude mice (4–6 weeks old) were subcutaneously injected in the armpit with 1 × 10 [7] stably expressed SKOV3 cells (n = 7 per group). Tumor width and length were measured weekly for 5 consecutive weeks. The tumor volumes were calculated according to the following formula: volume (mm [3]) = (length × width [2]) / 2. Five weeks later, the mice were euthanized and the tumors were removed. A portion of tumor tissues was extracted for follow-up immunohistochemistry.

### Intraperitoneal metastasis and bioluminescence imaging

SKOV3 cells that stably express luciferase vectors (luc-SLC25A21-AS1 or luc-PTBP3) were selected and stable cells (1 × 10 [6]) were used for the intraperitoneal injection of 4–6-week-old mice. Four weeks after the injection, the mice were anesthetized using isoflurane and injected with d-luciferin (Yeason, Shanghai, China). Metastases in the abdominal cavity of mice were captured by fluorescent signals using an IVIS Spectral in vivo Imaging System (Xenogene, Caliper Life Sciences). Images were analyzed using Live Image 4.1 software (Chromogen, Caliper Life Sciences).

### Immunohistochemistry assay

For antigen retrieval, deparaffinized sections were placed in a retrieval box to be heated. During this process, the buffer should be prevented from over-evaporating, and the sections should not be dried. A 5% BSA blocking solution was added dropwise, the tissue was evenly covered and sealed. After the primary antibody was added, sections were incubated overnight. The next day, the secondary antibody was added to tissues. Chromogen application was performed according to the manufacturer’s instructions, and the positive reaction was stained brownish yellow. Finally, the nuclei were counterstained with hematoxylin, dehydrated, and mounted onto slides.

### Clinical Specimens

Epithelial ovarian cancer tissues and the normal ovarian epithelial tissues with pathologic confirmation were collected from September 2019 to May 2022 at Women’s Hospital Zhejiang University School of Medicine (Hangzhou, China). All samples and clinical information were obtained the approvalof the Hospital Ethical Committee. In total, 30 normal epithelial ovarian tissues and 24 epithelial ovarian cancer tissues were subjected for qRT-PCR analysis. The information of all specimens is shown in Table S3. All samples were stored at -80°C until use.

### Statistical analysis

Statistical analyses were carried out using GraphPad Prism 8.0 (GraphPad Software, USA) and SPSS Statistics 20.0 (IBM, USA). Data conforming to normal distribution were presented as the means ± standard deviations (SDs). Data between groups were analyzed using the t-test. Otherwise, data are presented as the median ± interquartile range, using the Mann–Whitney test for comparisons. Differences were considered statistically significant at *P* < 0.05.

## Results

### Identification of SLC25A21-AS1, a lncRNA implicated in the development of EOC

**Fig. 1:**
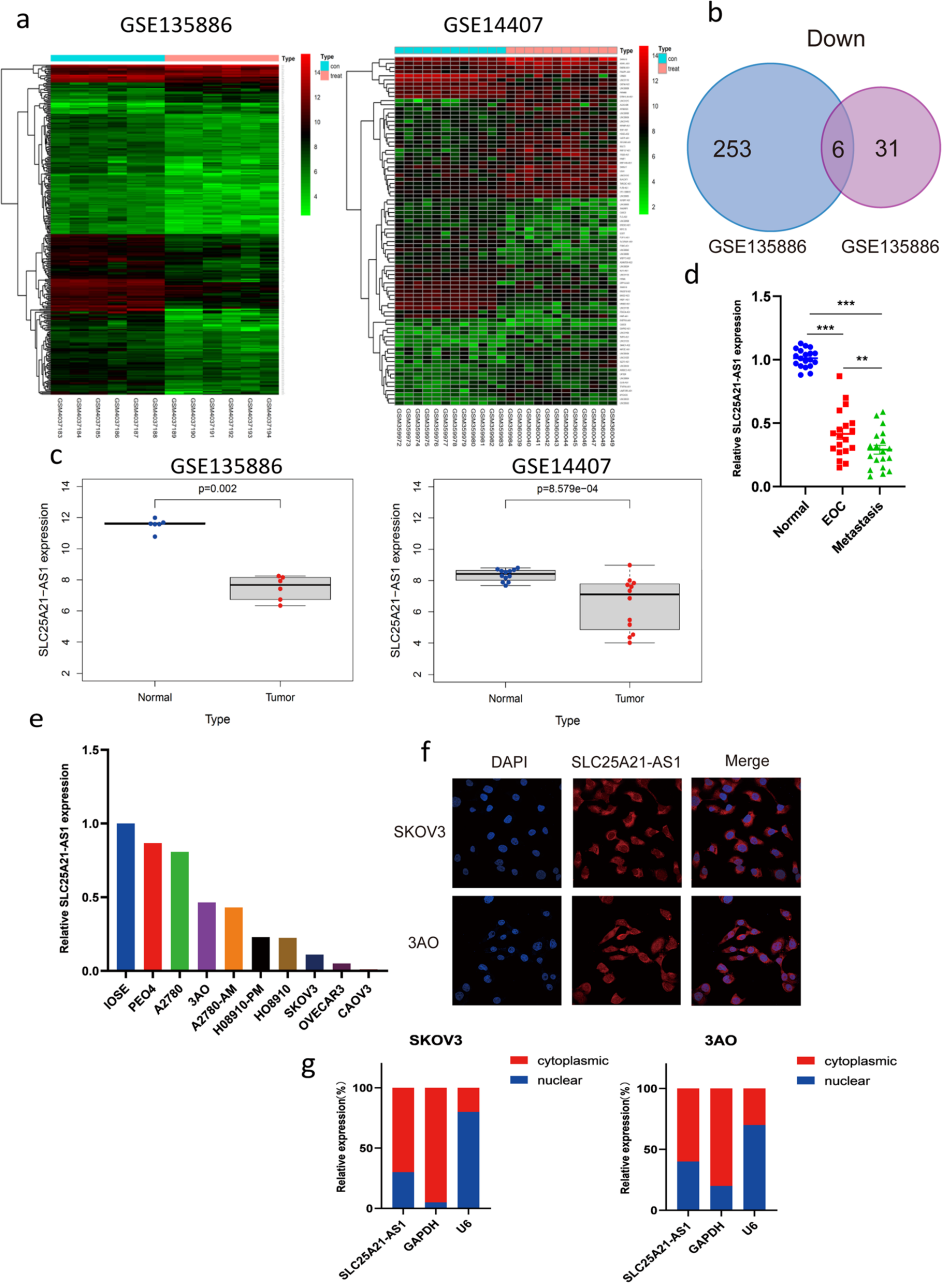
Discovery and identification of SLC25A21-AS1. **a** Screening according to GSE135886 and GSE14407. **b** Intersection of down-regulated genes in the two datasets. **c** SLC25A21-AS1 showed low expression in tumors in both datasets. The clinical data analysis was identified by package Limma using R software (version3.6.2). **d** Validation in tissue showed that the expression of SLC25A21-AS1 in EOC tissues and even metastatic tissues was significantly lower than that in normal ovarian tissue. **e** Validation of SLC25A21-AS1 expression levels in different cell lines. According to the difference in expression, two cell lines with moderate expression, SKOV3 and 3AO, were selected. **f** FISH assay were performed to verify the localization of SLC25A21-AS1 in EOC cells. SLC25A21-AS1 is labeled in both the nucleus and cytoplasm, but more in the cytoplasm. Blue represents nuclei and red represents SLC25A21-AS1. **g** Nucleocytoplasmic separation experiments confirmed the localization relationship of SLC25A21-AS1 in EOC cells. Error bars are the mean ± SEM of three independent replicate experiments. ***P<0.001

Differential gene expression (DGE) analysis was performed on normal and serous EOC samples using the GSE135886 and GSE14407 sample libraries (Fig. 1a). The DGE analysis revealed 37 upregulated and 259 downregulated lncRNAs in GSE135886, and 47 upregulated and 37 downregulated lncRNAs in GSE14407 in serous EOC samples (Fig. 1b). To narrow down the lncRNAs that have an impact on the occurrence and development of epithelial serous EOC, the results were filtered repeatedly to identify overlapping differentially expressed genes in the two sample libraries. This led to the identification of six co-downregulated lncRNAs: SLC25A21-AS1 (Fig. 1c), LINC00842, HHIP-AS1, LINC00908, PWAR6, and LINC00909 (Supplementary Fig. S1a). At the cellular level, only SLC25A21-AS1 was found to be expressed at a low level in EOC cells, consistent with expectations in normal ovarian cells and the description in GEPIA (Supplementary Fig. S1b). In contrast, the expression of LINC00842, HHIP-AS1, LINC00908, PWAR6, and LINC00909 in EOC and metastasis tissues did not meet expectations (Supplementary Fig. S1c). Therefore, we focused on this lncRNA. Next, we verified the differential expression levels of SLC25A21-AS1 in normal ovarian tissues, EOC in situ tissues, and distant metastasis tissues of EOC. In 20 pairs of tissue specimens, the content of SLC25A21-AS1 in EOC in situ tissues was significantly lower than that in normal ovarian tissues, and more so in distant metastatic tissues (Fig. 1d). These results motivated us to further study the expression of SLC25A21-AS1.

SLC25A21-AS1 expression was detected in nine currently recognized EOC cell lines (Fig. 1e). Subsequent experiments were performed in two of these cell lines (SKOV3 and 3AO). FISH assay revealed that SLC25A21-AS1 was enriched in both the nucleus and cytoplasm (predominantly) of the SKOV3 cells. In addition, about 70% of SLC25A21-AS1 is present in the cytoplasm, although a small portion is also present in the nucleus. (Fig. 1f). These observations were verified using nucleocytoplasmic separation experiments (Fig. 1g), which provided further understanding of the SLC25A21-AS1 expression.

### SLC25A21-AS1 suppresses the proliferation and metastatic abilities of EOC cells

**Fig. 2:**
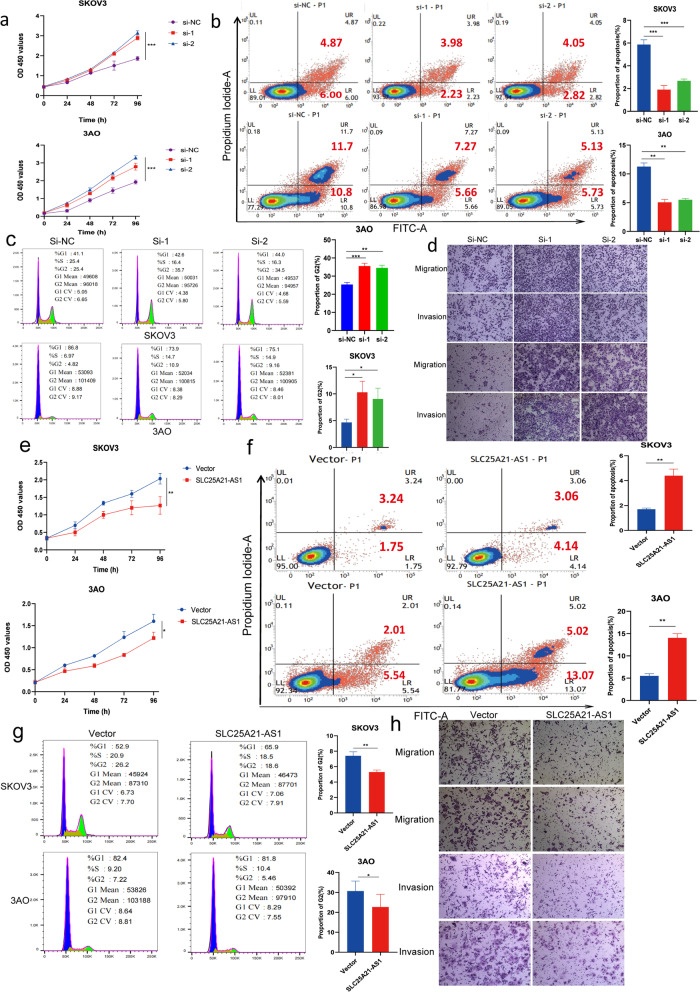
Functional effects of regulation of SLC25A21-AS1 in EOC cells. **a** Increased proliferation of EOC cells within 96 hours after knockdown of SLC25A21-AS1. ***P<0.001. Data represent mean ± SD (*n* = 3). **b** Reduced apoptosis of ovarian cancer cells after knockdown of SLC25A21-AS1. The effect on apoptosis compared through the number of early apoptotic process cells. Error bars are the mean ± SD of three independent replicate experiments. ***P<0.001. **c** The percentage of cells in G2 phase of ovarian cancer cells increased after knockdown of SLC25A21-AS1 (*n*=3). d. Knockdown of SLC25A21-AS1 can promote migration and invasion (*n*=3). The blue staining part is the positive count part. **e** Attenuated EOC cells proliferation within 96 hours after overexpression of SLC25A21-AS1. **P<0.01, *P<0.05. **f** Overexpression of SLC25A21-AS1 promotes apoptosis of EOC cells. Error bars values are all mean ± SD from three independent replicate experiments. **g** Overexpression of SLC25A21-AS1 decreased the percentage of cells in G2 phase (*n*=3). **h** Overexpression of SLC25A21-AS1 inhibited the migration and invasion of EOC cells (*n*=3)

To clarify the contribution thereof to the occurrence and development of EOC, we designed three siRNAs for targeting SLC25A21-AS1, and selected the two most potent siRNAs. This ensured effective knockdown of SLC25A21-AS1 in the selected cell lines (Supplementary Fig. S2b). Concurrently, an overexpression plasmid was designed for this lncRNA to achieve its overexpression (Supplementary Fig. S2d). Interestingly, CCK8 proliferation experiments for the SLC25A21-AS1-knockdown SKOV3 and 3AO cells revealed that knockdown of SLC25A21-AS1 significantly increased proliferation (Fig. 2a). Apoptosis experiments demonstrated that the knockdown of SLC25A21-AS1 expression impaired apoptosis in EOC cells to some extent (Fig. 2b). Cell cycle experiments showed that when SLC25A21-AS1 expression is knocked down, a significantly higher proportion of cells in G2 phase was noted, indicating increased cell division (Fig. 2c). A Transwell assay was used to assess the potency of SLC25A21-AS1 on the metastasis of EOC cells. We realized that knocking down of SLC25A21-AS1 expression increased the migration ability of EOC cells, which promoted invasion (Fig. 2d). Conversely, cells overexpressing SLC25A21-AS1 showed significantly reduced proliferation (Fig. 2e). At the same time, high expression of SLC25A21-AS1 resulted in reversal a small percentage of apoptosis in EOC cells. (Fig. 2f). Consistently, EdU experiments revealed that EdU signals clearly improved when SLC25A21-AS1 expression was knocked down (Supplementary Fig. S2c) and declined in SLC25A21-AS1-overexpression cells (Supplementary Fig. S2e). Overexpression of this molecule resulted in fewer cells in the G2 phase and less active division (Fig. 2g).

The opposite was observed when SLC25A21-AS1 was overexpressed (Fig. 2h). Next, we verified the effect of knockdown and overexpression of SLC25A21-AS1 expression compared with its host gene, SLC25A21, and found that SLC25A21-AS1 knockdown or overexpression had no effect on SLC25A21 expression levels (Supplementary Fig. S2a). This indicates that SLC25A21-AS1 has a function in the occurrence and development of EOC cells independent of the role of its host RNA.

### SLC25A21-AS1 binds to PTBP3

**Fig. 3:**
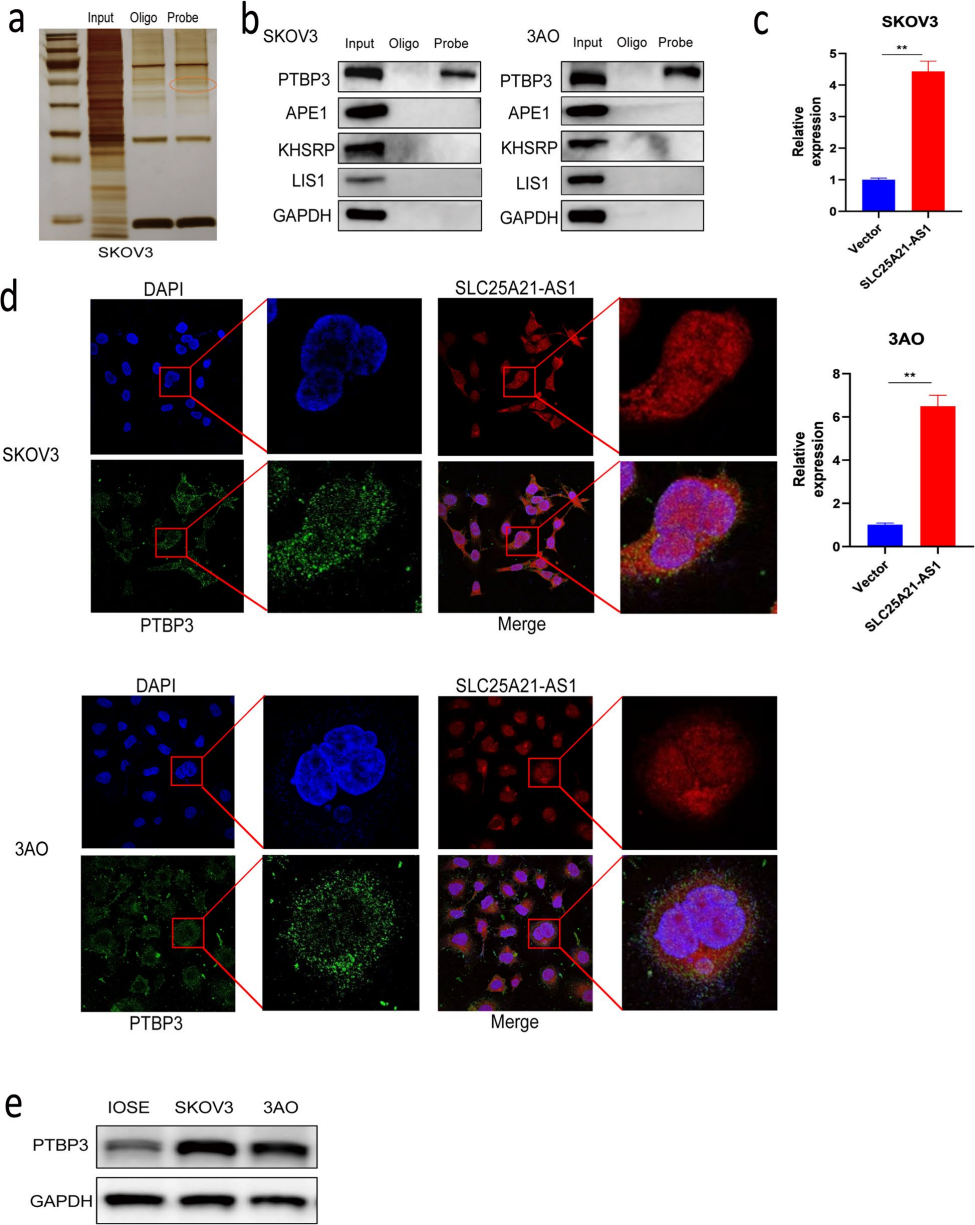
The RNA-binding protein PTBP3 was screened and identified. **a** RNA pull-down experiments and silver staining to find specific bands. Specific band is marked in orange circles. **b** RNA-pulldown assay to identify candidate proteins from MS analysis. **c** RIP experiments confirmed that PTBP3 was better enriched when SLC25A21-AS1 was overexpressed (n=3). *P<0.05. **d** IF-FISH assay confirmed the co-localization between SLC25A21-AS1 and PTBP3, and PTBP3 was mainly present in the cytoplasm, but also partly in the nucleus. Blue represents the nucleus, red represents SLC25A21-AS1, and green represents PTBP3. **e** The expression of PTBP3 in EOC cells was higher than that in normal ovarian cells

To further clarify the role of SLC25A21-AS1 in EOC, a pull-down experiment was performed. The resulting proteins were separated using SDS-PAGE and silver stained (Fig. 3a, S4a). Specific bands were cut from the gel and detected by protein profiling three times. Based on the scores and unique peptides of the protein profiles (Table S2), four potential RNA-binding proteins which had the top scores were identified. They included PTBP3, APE1, KHSRP, and LIS1. We were able to verify the interaction of SLC25A21-AS1 with PTBP3, but not with the remaining proteins (Fig. 3b). This interaction was further confirmed in RIP experiments on PTBP3 (Fig. 3c, S3a) and IF-FISH co-localization experiments (Fig. 3d, localization of PTBP3 in cells is represented in green). The latter showed roughly complete colocalization of PTBP3 and SLC25A21-AS1, and that PTBP3 was present in both the nucleus and cytoplasm of SKOV3 and 3AO cells. Moreover, nucleocytoplasmic separation experiments, performed for PTBP3, verified these findings (Supplementary Fig. S3b). The role of PTBP3 in EOC is unknown; hence, we examined the expression of PTBP3 in EOC cells and found that PTBP3 in SKOV3 and 3AO cells was highly expressed compared with its expression in normal ovarian cells (Fig. 3e), consistent with the results in GEPIA (Supplementary Fig. S4b).

### PTBP3 promotes the development of EOC cells

A detailed functional investigation of PTBP3 was conducted to reveal the role of PTBP3 in EOC. Three specific siRNAs and an overexpression plasmid were designed and constructed for PTBP3. Two of the three siRNAs with the best knockdown efficiency were selected to ensure experimental accuracy (Supplementary Fig. S4c) and selected the overexpression efficiency after overexpression of PTBP3 (Supplementary Fig. S4e).

**Fig. 4:**
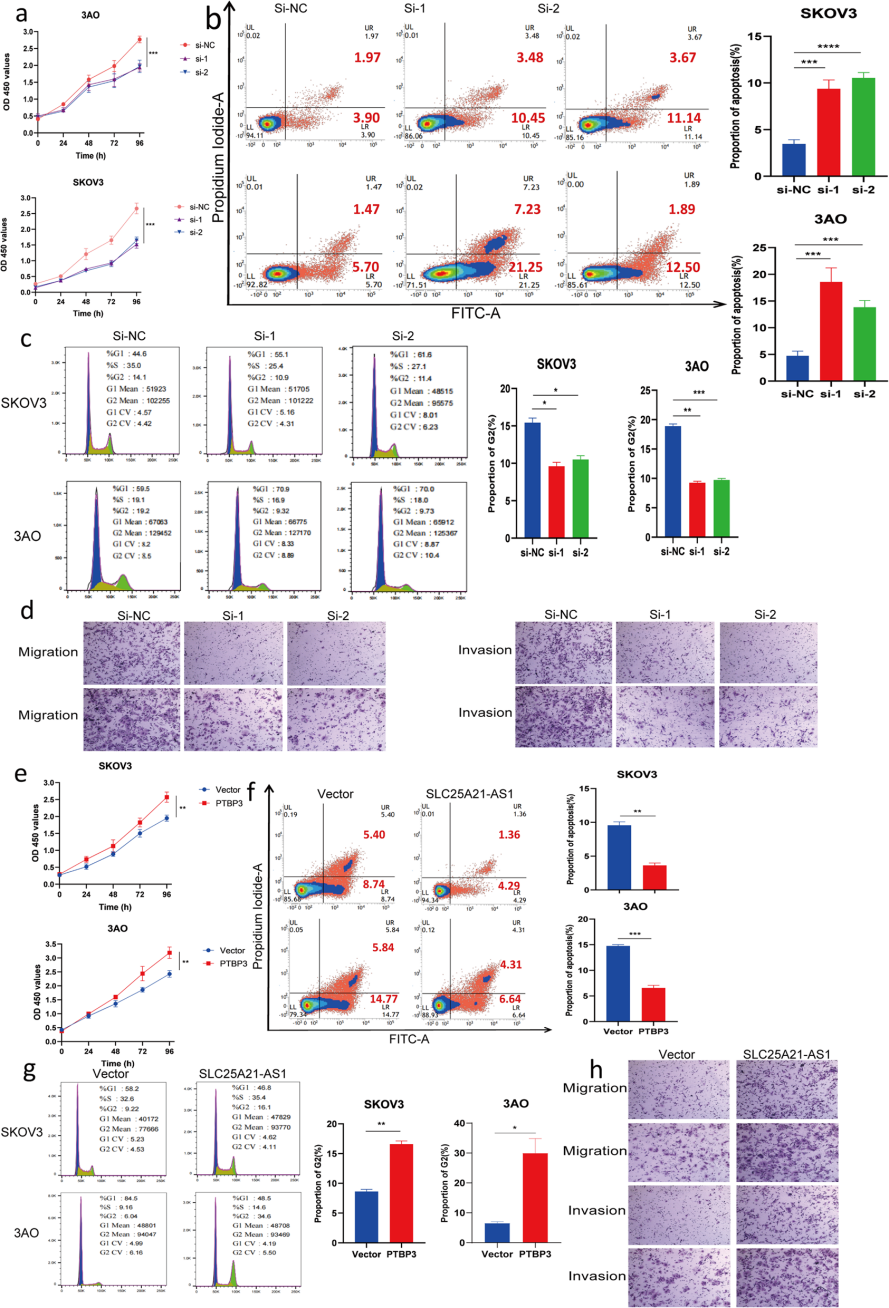
The function of ovarian cancer cells by regulating PTBP3. **a** After knocking down PTBP3, the proliferation rate of EOC cells was slowed down within 96 hours. Data are mean ± SD from three independent replicate experiments. ***P<0.001. **b** Increased apoptosis of EOC cells after knockdown of PTBP3. Error bars represent the mean ± SD of three independent replicates. **c** The percentage of cells in G2 phase decreased after knockdown of PTBP3 (*n*=3). **d** The migration and invasion rates of ovarian cancer cells were decreased after knockdown of PTBP3 (*n*=3). **e** The proliferation of ovarian cancer cells was significantly accelerated after overexpression of PTBP3. **P<0.01. **f** Overexpression of PTBP3 significantly reduced apoptosis. Error bars represent the mean ± SD of three independent replicates. ***P<0.001, **P<0.01.**g** The percentage of EOC cells in G2 phase was significantly increased after overexpression of PTBP3 (*n*=3). **h** The migration and invasion of ovarian cancer cells were significantly increased after overexpression of PTBP3, data represent mean ± SD. **P<0.01

The CCK8 experiments suggested that knockdown of PTBP3 expression reduced EOC cell proliferation; the EDU experiment further supported this change (Fig. 4a, S4d). Conversely, PTBP3 overexpression significantly accelerated EOC cell proliferation (Fig. 4e, S4f). The results from the apoptosis experiments demonstrated that reduced PTBP3 expression increased apoptosis (Fig. 4b), while increased PTBP3 expression decreased apoptosis (Fig. 4f). Moreover, changes in PTBP3 expression during the cell cycle altered the percentage of cells in G2 phase: when PTBP3 expression was low, the percentage decreased (Fig. 4c), and vice versa (Fig. 4g).

In metastasis-related experiments, migration and even invasion abilities in vitro were clearly enhanced due to PTBP3 overexpression, while PTBP3 knockdown slowed the transfer rate (Fig. 4d, h). These functional experiments revealed that PTBP3 promotes the occurrence and development of EOC.

### SLC25A21-AS1 specifically induces PTBP3 degradation via the ubiquitin–proteasome pathway

**Fig. 5:**
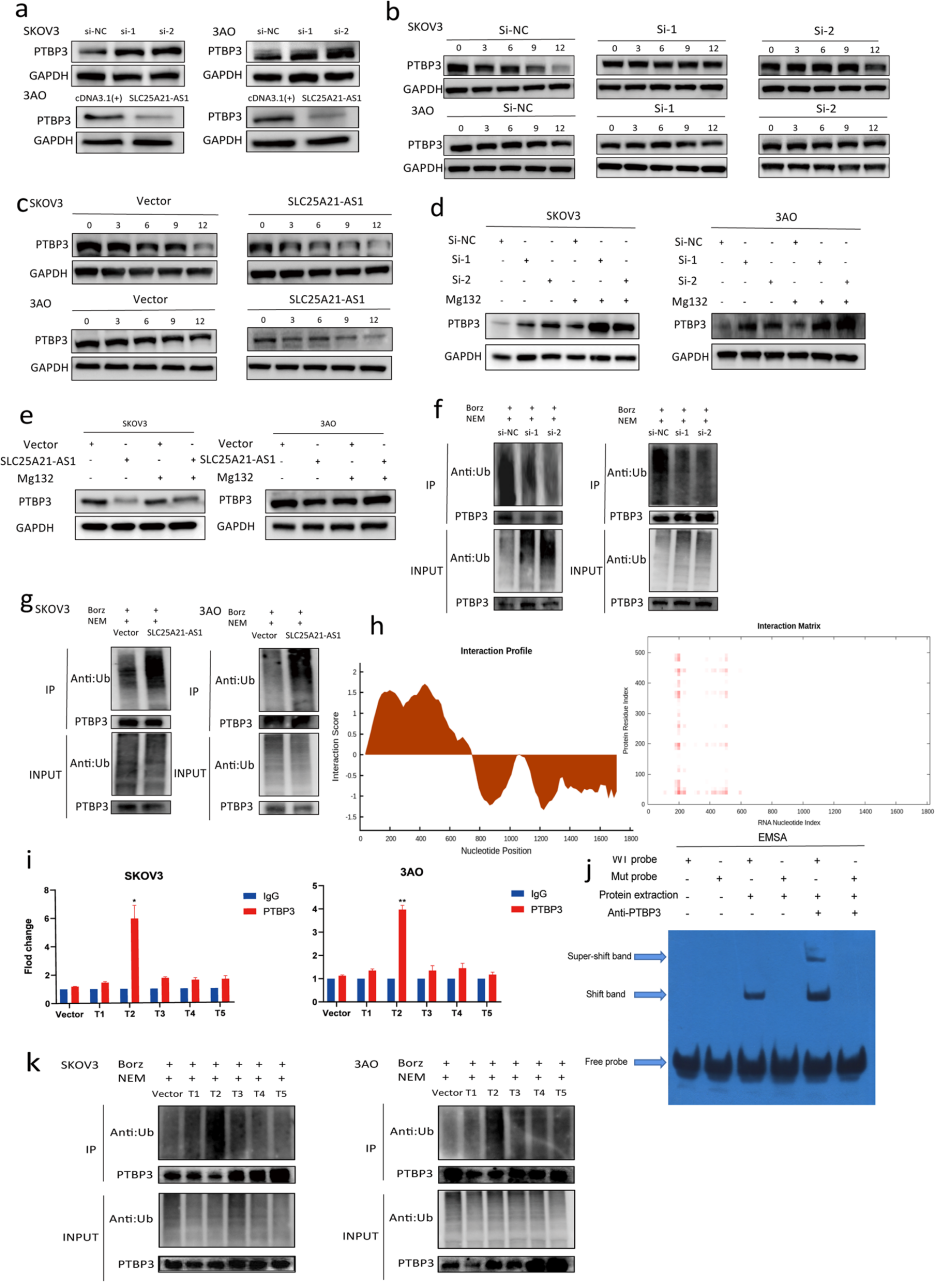
SLC25A21-AS1 directly regulates PTBP3. **a** The effect of knockdown or overexpression of SLC25A21-AS1 on the expression of PTBP3 was detected by Western blot (WB). **b-c** Observe the protein degradation within 12 hours after adding CHX. **d-e** After knockdown or overexpression of SLC25A21-AS1 and adding proteasome inhibitor (Mg132), the stability of PTBP3 was increased compared with the control group. **f-g** Ubiquitination assay to detect the ubiquitination level of PTBP3 in SLC25A21-AS1 knockdown or overexpressing EOC cells. Borz (250nM) and NEM (5μm) were added for 6h. **h** Using catRAPID to predict the binding sites of PTBP3 and SLC25A21-AS1. **i** The enrichment between PTBP3 and different truncations was detected by RIP assay. The enrichment of T2 was significantly better than that of other truncations (*n*=3). **P<0.01, *P<0.05. **j** Electrophoretic mobility shift analysis (EMSA) to verify the interaction between T2 truncation and its mutants with PTBP3. **k** Ubiquitination assay between different truncations and PTBP3. Borz (250nM) and NEM (5μm) were added for 6h

To explore the mechanism behind the SLC25A21-AS1 binding to PTBP3, a series of follow-up experiments were performed. Western blot assays revealed that the PTBP3 protein level was enhanced until the amount of SLC25A21-AS1 declined, while it was reduced when SLC25A21-AS1 showed increased expression (Fig. 5a, S5a). Interestingly, alternating the expression of SLC25A21-AS1 did not affect PTBP3 mRNA levels (Supplementary Fig. S5b). Moreover, altered expression of PTBP3 had no effect on SLC25A21 expression (Supplementary Fig. S5c). These outcomes suggest that SLC25A21-AS1 directly adjusts PTBP3 protein levels after binding to it, rather than using it as a parent or bridge to regulate the expression of its downstream molecules. To determine whether PTBP3 expression in EOC is regulated through ubiquitination, cycloheximide was used to verify protein stability. After 12 h of incubation, we found that the half-life of PTBP3 in the SLC25A21-AS1-knockdown cells was significantly enhanced, while its stability in the SLC25A21-AS1-overexpression group was significantly decreased (Fig. 5b, c). This indicates that SLC25A21-AS1 regulates PTBP3 expression by promoting protein degradation.

Next, we treated the SKOV3 and 3AO cells with the proteasome inhibitor MG132. We found that regardless of the knockdown or overexpression of SLC25A21-AS1, MG132 treatment weakened protein degradation, resulting in protein levels being more stable compared with the control group (Fig. 5d,e). Moreover, immunoprecipitation results showed that ubiquitin in SLC25A21-AS1-knockdown cells treated with NEM (inhibitor of endogenous deubiquitinase) and Borz (non-selective inhibitor of deubiquitinase) had a lower degree of ubiquitination, compared with SLC25A21-AS1-overexpressing cells treated with the same inhibitors (Fig. 5f, g). These results suggest that SLC25A21-AS1 directly regulates PTBP3 expression via the ubiquitin-proteasome pathway.

To further examine the interaction between SLC25A21-AS1 and PTBP3, we used catRapid to predict the potential PTBP3 binding site on the SLE25A21-AS1 sequence. (Fig. 5h). In silico prediction results (Supplementary Fig. S6a) showed the full-length SLC25A21-AS1 could be divided into five segments and truncated bodies were formed (Supplementary Fig. S6b, c). Moreover, RIP experiments revealed that the T2 segment was enriched for PTBP3, and that the addition of the negative control group ruled out the binding error of endogenous SLC25A21-AS1 to a certain extent (Fig. 5i). To ensure accuracy, we tested the amplification products of T1–T5 twice to verify the sequence consistency of the amplified fragments and the truncated body (Supplementary Fig. S7a). The EMSAs confirmed the binding of the T2 segment to PTBP3. After Mut T2, the PTBP3 was not enriched, and the super-shift band did not appear after the addition of the antibody, confirming PTBP3 binding to SLC25A21-AS1 in the T2 segment (180–256 bp) (Fig. 5j).

To determine whether the ubiquitin-proteasome pathway mechanism of SLC25A21-AS1 and PTBP3 is closely related to the association between the two, we performed immunoprecipitation experiments with 5-segment truncations and used control cell groups to exclude endogenous interference. The differences in ubiquitination between the groups were analyzed, and it was found that the level of ubiquitination in the T2 segment was significantly increased, while ubiquitination in the other segments was similar to the negative control group (Fig. 5k).

### PTBP3 reverses the role of SLC25A21-AS1 in EOC

**Fig. 6:**
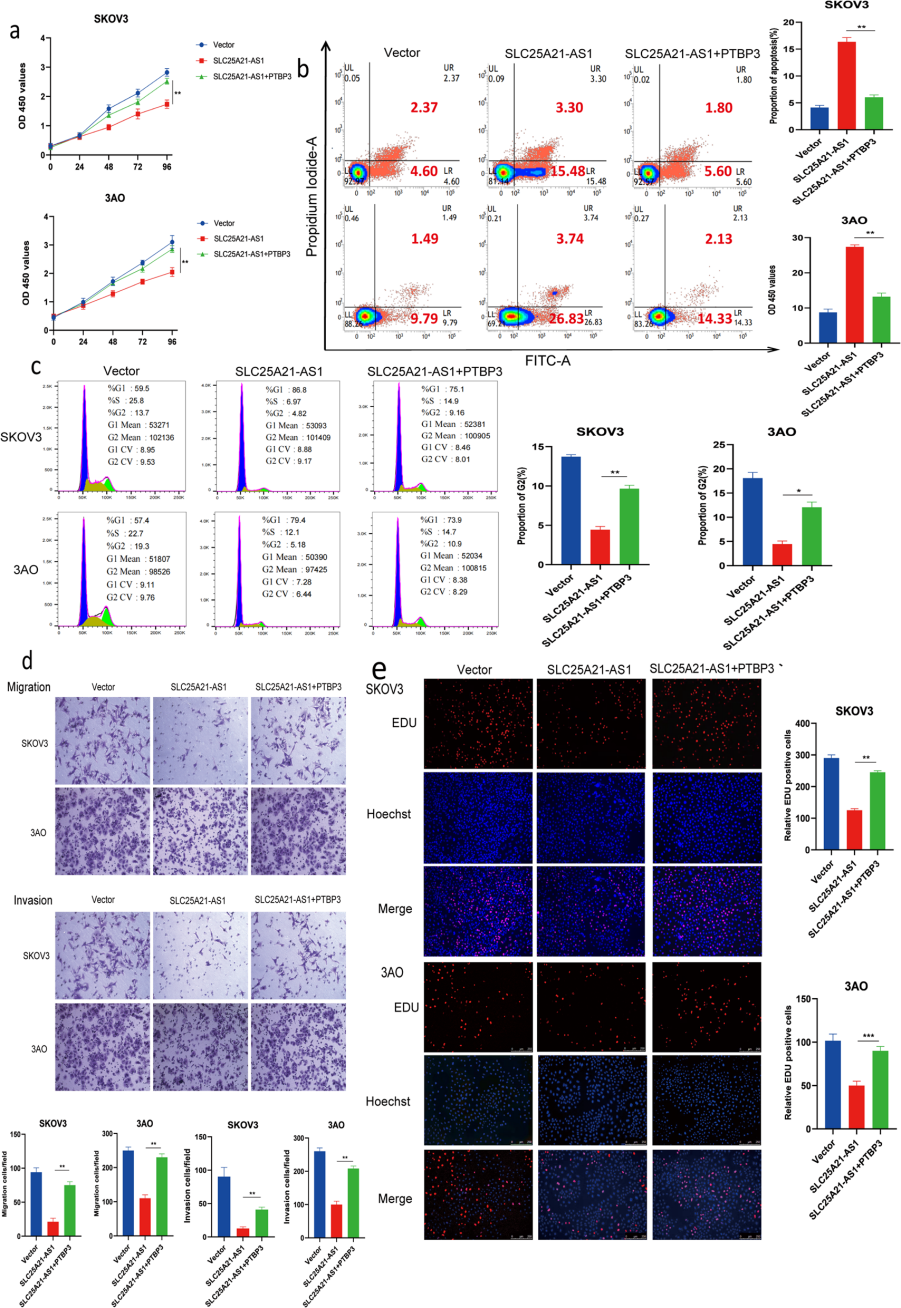
PTBP3 can reverse the inhibitory effect of SLC25A21-AS1 on EOC cells. **a** PTBP3 rescued the inhibitory effect of SLC25A21-AS1 on EOC cells proliferation (*n*=3). **b** Overexpression of both SLC25A21-AS1 and PTBP3 reduced the apoptosis of EOC cells compared with the SLC25A21-AS1 group**P<0.01. **c** The addition of PTBP3 with SLC25A21-AS1 re-increased the percentage of ovarian cancer cells in G2 phase. **d** PTBP3 effectively rescued the inhibitory migration and invasion effects of SLC25A21-AS1 (*n*=3). **e** EdU assay to detect the role of SLC25A21-AS1 and PTBP3 in ovarian cancer cell proliferation. Error Bars represent the mean ± SD of three independent replicate experiments. ***P<0.001, **P<0.01

To determine whether PTBP3 mediates the role of SLC25A21-A1 in serous OC, we overexpressed SLC25A21-AS1 stably in SKOV3 and 3AO cells (Supplementary Fig. S8a) and expressed PTBP3 in cells from one of the groups. The CCK8 assay revealed that simultaneous overexpression of SLC25A21-AS1 and PTBP3 alleviated the inhibitory effect on cell proliferation, compared to SLC25A21-AS1 expression alone (Fig. 6a). In keeping with this, the EdU experiment showed that, compared with the SLC25A21-AS1 overexpression group, there was a significant improvement in the proliferation rate in the SLC25A21-AS1 and PTBP3 overexpression group (Fig. 6e). Moreover, overexpression of PTBP3 effectively rescued SLC25A21-AS1-induced apoptosis (Fig. 6b), with an increased percentage of cells in the G2 phase (Fig. 6c). Furthermore, after the addition of PTBP3, SLC25A21-AS1 alleviated the consistency of migration and invasion (Fig. 6d). These outcomes denote that PTBP3 can reverse the inhibitory effect of SLC25A21-AS1 in EOC cells.

### SLC25A21-AS1 inhibits tumor growth and metastasis in a xenograft model

**Fig. 7:**
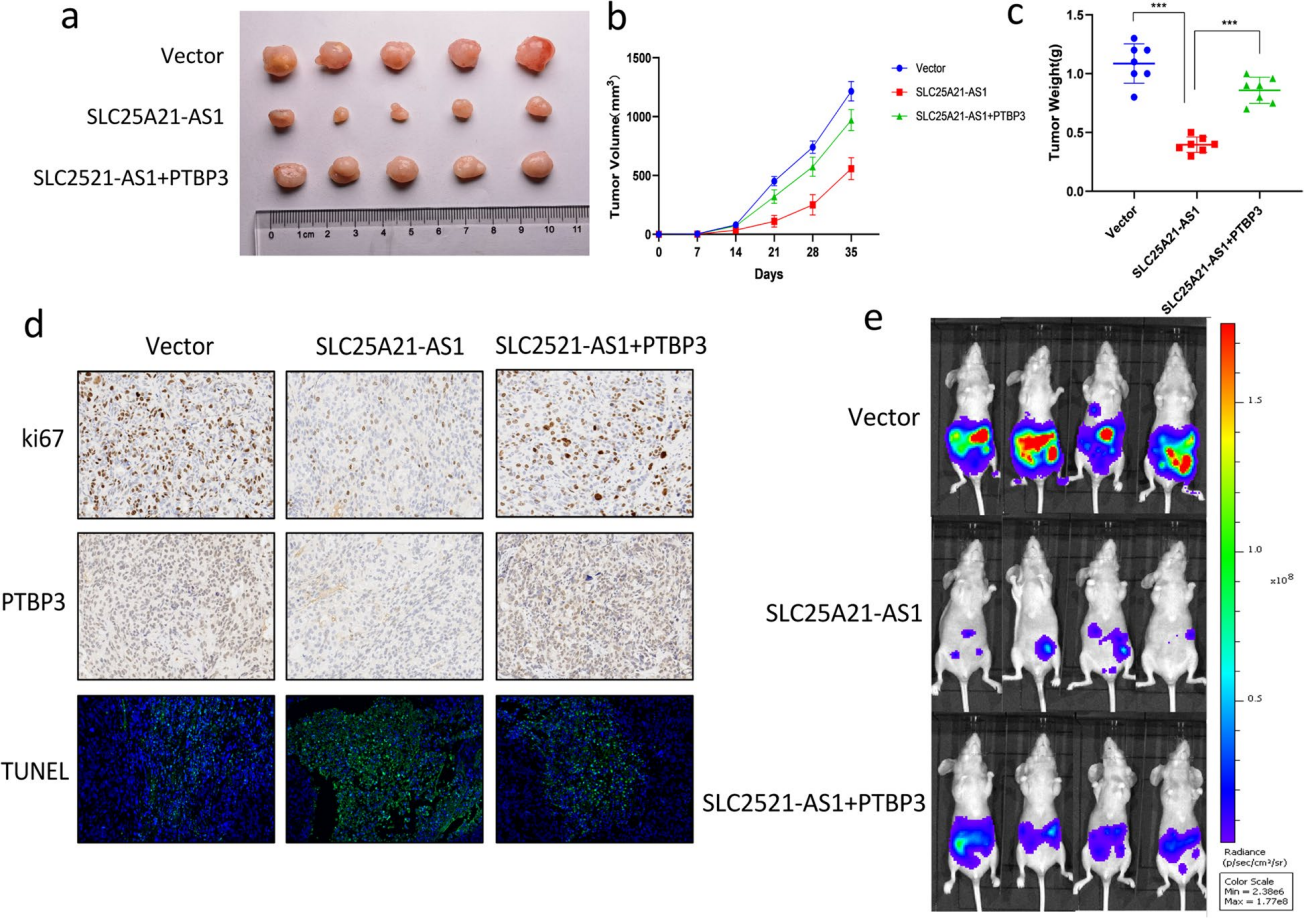
Inhibition of SLC25A21-AS1 and reversal of PTBP3 in xenograft tumor models. **a** SKOV3 cells stably expressing SLC25A21-AS1, both SLC25A21-AS1 and PTBP3 or empty vectors were subcutaneously injected into nude mice, each mouse was injected with 1x107 cells, 35 days later, the nude mice were euthanized and the tumors were completely removed and photographed. **b** Tumor volume (ab2/2) was measured every 6 days from seeding of stably expressing cells. **c** Tumor weight on the day after euthanizing nude mice. Datas represent mean ± SEM (*n*=7). **d** H&E staining and immunohistochemistry (IHC) were used to detect the proliferation, apoptosis and relative protein levels of PTBP3 in different groups. Ki67 shows proliferation levels and TUNEL shows apoptosis. Scale bars=100um. **e** Using an in vivo bioluminescence imaging system to detect tumor metastasis in different groups. Representative images (*n*=7) are shown

After clarifying the in vitro effect of SLC25A21-AS1, we sought to confirm its effect in vivo. For this purpose, we subcutaneously injected different groups of SKOV3 stably transfected cells (Supplementary Fig. S8a) into nude mice, and periodically measured the tumor sizes to observe tumor proliferation after formation. The results showed that the SLC25A21-AS1 overexpression group significantly inhibited tumor growth, while tumors in the PTBP3 group were larger (Fig. 7a, b). Notably, tumors were lighter in the SLC25A21-AS1 group than the other two groups (Fig. 7c). The tumor tissues were removed and used for RT-qPCR. In order to ensure the accuracy of the experiment, we re-extracted RNA and protein from tumors dissected from nude mice and detected the expression levels of SLC25A21-AS1 and PTBP3 (Supplementary Fig. S8c, d). The results were consistent with our assumptions. Immunohistochemistry indicated that proliferation in the SLC25A21-AS1 group was weaker but recovered in the presence of PTBP3. In addition, apoptosis in the SLC25A21-AS1 group increased compared with the control group, while PTBP3 reversed this effect (Fig. 7d). In addition, three groups of nude mice were selected for intraperitoneal injection to observe tumor metastasis. After four weeks of observation, distinct patterns were observed in the motility in among different groups of nude mice (Fig. 7e). Motility in the SLC group was, as expected, slower than the control group; addition of PTBP3 restored tumor metastasis to a certain extent, in agreement with the in vitro results.

### SLC25A21-AS1 acts as a prognostic protective molecule in various tumors

**Fig. 8:**
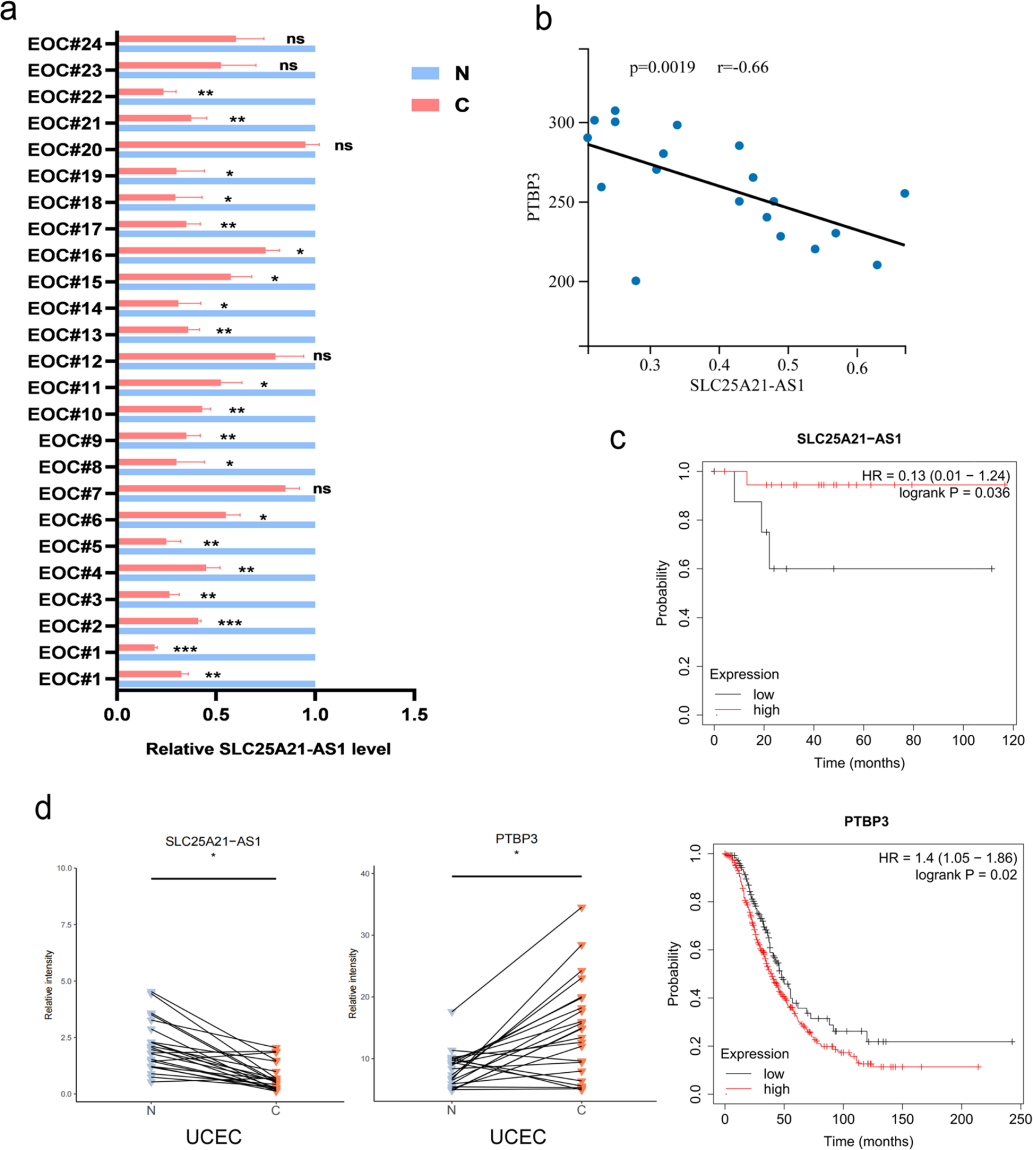
Clinical significance of SLC25A21-AS1. **a** The tumor tissues and adjacent normal tissues of 24 EOC patients were randomly selected, and the relative expressions of SLC25A21-AS1 in different groups were detected by real-time quantitative PCR. **b** Correlation analysis of SLC25A21-AS1 and PTBP3.The data were based on 19 patients with a decline in SLC25A21-AS1 in a. **c** Clinical survival analysis on SLC25A21-AS1 and PTBP3. The significance of the relationship was tested by the log-rank test. **d** Differences in relative expression levels of SLC25A21-AS1 and PTBP3 in patients with endometrial carcinoma (UCEC). The Wilcoxon rank sum test in R was used to statistically test the expression levels of target genes in target cancer samples, and set paired = TRUE. *P<0.05

We randomly selected 24 patients with EOC and verified the expression of SLC25A21-AS1 in their tumor or paracancerous tissues. The results demonstrated that the levels of SLC25A21-AS1 in tumor tissues of 19 patients were significantly reduced compared with those in paracancerous tissues. No significant differences were observed in the other 5 patients (Fig. 8a). At the same time, we performed the correlation analysis between SLC25A21-AS1 and protein PTBP3 in 19 patients with a statistically significant reduction in SLC25A21-AS1 and found a negative correlation between them (Fig. 8b). Besides, We plotted correlation analysis in the TCGA database using expression data of SLC25A21-AS1 and PTBP3 mRNA. The results of R [2]=0.00108 indicated that SLC25A21-AS1 had no obvious correlation with the mRNA of PTBP3. This result was also consistent with the verification results of figure S5b mentioned above, which further confirmed the direct regulatory relationship between SLC25A21-AS1 and PTBP3 protein (Supplementary Fig. S9a). In order to detect the difference of protein PTBP3 in clinical specimens, we performed immunohistochemical staining with 19 patient tissues with significantly reduced SLC25A21-AS1 in EOC of Fig. 8a. The staining results showed that except for the PTBP3 protein in the EOC tissue of patient #22, PTBP3 protein in the EOC group had obvious staining in the remaining 18 pairs, and this finding confirmed the high expression of PTBP3 in EOC tumor tissues (Fig. S9b). Moreover, bioinformatics analysis identified SLC25A21-AS1 as a potential clinical prognostic protective molecule, with a great impact on the survival rate of patients with EOC. While PTBP3 the presence of PTBP3 leads to a decrease in patient survival and an increase in fatality (Fig. 8c). To verify whether SLC25A21-AS1 has clinical significance in other tumors, we performed TCGA analysis on more than 20 tumors and found that, in other gynecological tumors, such as Uterine Corpus Endometrial Carcinoma (UCEC), SLC25A21-AS1 expression was low, whereas PTBP3 expression was high (Fig. 8d). Meanwhile, SLC25A21-AS1 expression in other systemic tumors was lower than that in normal tissues (Supplementary Fig. S9c, S9d), consistent with the description in GEPIA (Supplementary Fig. S1). Taken together, these results indicate that SLC25A21-AS1 can function as a potential clinical inhibitory molecule in EOC, and may serve as a therapeutic target in various tumors.

## Discussion

Ovarian cancer has the high mortality rate among gynecological cancers worldwide [27]. As the most common type of OC, early diagnosis of EOC is challenging, with many patients already in the middle and late stages during diagnosis. Very few therapeutic targets for EOC are known, leaving room for exploration [28, 29]. Unfortunately, EOC is still considered one of the most difficult tumors to manage due to its clinical, biological, and molecular complexity [30]. Current research is focused on deep molecular [31] and cellular analyses [32, 33]. However, few studies exist on EOC-specific treatments [34]. Achieving personalized precision medicine requires the identification of better biomarkers that will enable the selection of patients who will benefit from chemotherapy, targeted drugs, or immunotherapy [27]. In the present study, we discovered the lncRNA SLC25A21-AS1, which is located on chromosome 14, and revealed its roles in cycle arrest, apoptosis, proliferation, invasion, and migration of EOC cells.

The SLC25A family proteins have been extensively studied and many reports have revealed their links to tumors and metabolism [35, 36]. However, few studies have focused on SLC25A21-AS1 [37, 38], and its role in EOC remains unresolved. Typically, lncRNAs act via target molecules [39], such as lncPOP1-1, that promotes cisplatin resistance in squamous cell carcinoma, which occurs in the neck, by interacting with MCM5 [40]. In addition, lncCPLC promotes colorectal resistance by regulating cancer progression via interaction with ZBTB [41]. At the same time, some lncRNAs in OC have also been reported to control OC resistance [42] or autophagy. For example, by binding to the 3′-UTR of FOS-like 2 (FOSL2), miR-143 can negatively regulate FOSL2 expression, suggesting that the UCA1/miR-143 axis may have potential therapeutic value for the treatment of cisplatin resistance in ovarian cancer patients. Besides, according to the reports, RP11-135L22.1 can inhibit cisplatin-induced autophagy, thereby enhance the effect of cisplatin in OC [43]. However, there is no good target for the application of lncRNAs in OC at present, and the lncRNA that can affect the progression and prognosis of OC are still needed in clinical practice. In the present study, we demonstrated that SLC25A21-AS1 was widely presented in the nucleus and cytoplasm, with a more abundant accumulation in the cytoplasm, suggesting that it likely functions at the post-transcriptional level.

RNA pull-down assays revealed that SLC25A21-AS1 interacts with PTBP3. The latter has been acknowledged to play a part as a tumor-promoting factor in sundry cancers [22], with its aberrant expression having multiple effects on different cancers. For example, PTBP3 functions on mesothelial-mesenchymal transition by maintaining mRNA stability [44], and facilitates the growth and metastasis by transforming activation on HIF-1α [45]. It has been reported that SLC25A21-AS1 specifically binds to PTBP3 in the 180–256bp segment. When this site was mutated, the binding ability of SLC25A21-AS1 to PTBP3 was weakened. In addition, the ubiquitin–proteasome system is the main pathway of protein degradation in vivo, with 80% of proteins in the body being degraded through this pathway. In our study, ubiquitination of the truncated body confirmed that the binding of the two proteins is closely related to ubiquitination regulation. Consistent with previous reports, which showed that PTBP3 functions through ubiquitination [46], we demonstrated that SLC25A21-AS1 acts on the PTBP3 protein through the ubiquitin–proteasome pathway, and that this degradation of PTBP3 affects the proliferative and metastatic effects thereof in EOC. We showed that loss of PTBP3 expression inhibited EOC proliferation and metastatic processes, which is in keeping with the findings published by other groups [46–49]. Moreover, functional verification demonstrated that PTBP3 could reverse the inhibitory function of SLC25A21-AS1 in EOC and confirmed that SLC25A21-AS1 exerts its effects by regulating PTBP3 expression.

We further confirmed our findings in vivo and examined the low expression of SLC25A21-AS1 in tumors from 24 clinical patients. In the last part of the study, using bioinformatics analysis, we found that SLC25A21-AS1 has a better survival-promoting effect against EOC. Moreover, our study is not limited to EOC, because SLC25A21-AS1 is also expressed in other tumors, albeit at lower levels. Taken together, our results revealed that SLC25A21-AS1 likely plays a crucial part as an important tumor suppressor molecule in most tumors, and further research is required to confirm this finding. And as a direct regulator of SLC25A21-AS1, PTBP3 degradation can increase the inhibitory effect of SLC25A21-AS1 on EOC proliferation and metastasis to a certain extent. The poor prognosis of PTBP3 makes PTBP3 degradation become a new idea of molecular targeted therapy.

## Conclusions

Our research sheds new light on the significance of a lncRNA in EOC and provides evidence for its molecular mechanism in ubiquitination-mediated protein degradation. This is the first study to define SLC25A21-AS1 as a negative regulator of EOC occurrence and development. Mechanistically, SLC25A21-AS1 is involved in the progression of EOC by binding to the downstream protein PTBP3 and affecting its ubiquitination. We believe that SLC25A21-AS1 can potentially be employed as a good prognostic protective molecule in EOC.

## Supplementary Information

**Additional file 1.**

## Data Availability

The datasets and materials used and/or analyzed during the current study are available from the corresponding author on reasonable request.

## Abbreviations

EOC: Epithelial ovarian cancer
lncRNA: Long non-coding RNA
SLC25A21-AS1: SLC25A21 antisense RNA 1
OC: Ovarian cancer
HGP: Human Genome Project
Human Genome Project: Encyclopedia of DNA Elements
PTBP3: Polypyrimidine tract-binding protein 3
HnRNP: Heterogeneous nuclear ribonucleoproteins
MiR: MicroRNA
DMEM: Dulbecco's Modified Eagle Medium
BSA: bovine serum albumin
FISH: Fluorescence in situ hybridization
SiRNAs: Small interfering RNAs
UCEC: Uterine Corpus Endometrial Carcinoma
LUSC: Lung squamous cell carcinoma
STAD: Stomach adenocarcinoma
ESCA: Esophageal carcinoma
READ: Rectum adenocarcinoma
