# Correction: Long non-coding RNA SLC25A21-AS1 inhibits the development of epithelial ovarian cancer by specifically inducing PTBP3 degradation

**DOI:** 10.1186/s40364-024-00659-w

**Published:** 2024-09-27

**Authors:** Sihui Li, Shizhen Shen, Wanzhong Ge, Yixuan Cen, Songfa Zhang, Xiaodong Cheng, Xinyu Wang, Xing Xie, Weiguo Lu

**Affiliations:** 1 Women’s Reproductive Health Laboratory of Zhejiang Province, School of Medicine, Women’s Hospital, Zhejiang University, Hangzhou, 310006 China; 2 Cancer Center, Zhejiang University, Hangzhou, 310058 China; 3 Division of Human Reproduction and Developmental Genetics, Zhejiang Provincial Key Laboratory of Precision Diagnosis and Therapy for Major Gynecological Diseases, Women’s Hospital, Zhejiang University School of Medicine, Hangzhou, 310006 China; 4 Institute of Genetics, Zhejiang University, Hangzhou, 310058 China; 5 Department of Gynecologic Oncology, School of Medicine, Women’s Hospital, Zhejiang University, Hangzhou, 310006 China

**Biomarker Research (2023) 11:12**

10.1186/s40364-022-00432-x

The original article contains errors in Fig. 4 and Fig. 6 as well as some revision highlights in the supplementary material. The corrected figures + ESM can be viewed via this Correction article.

**Fig. 4:**
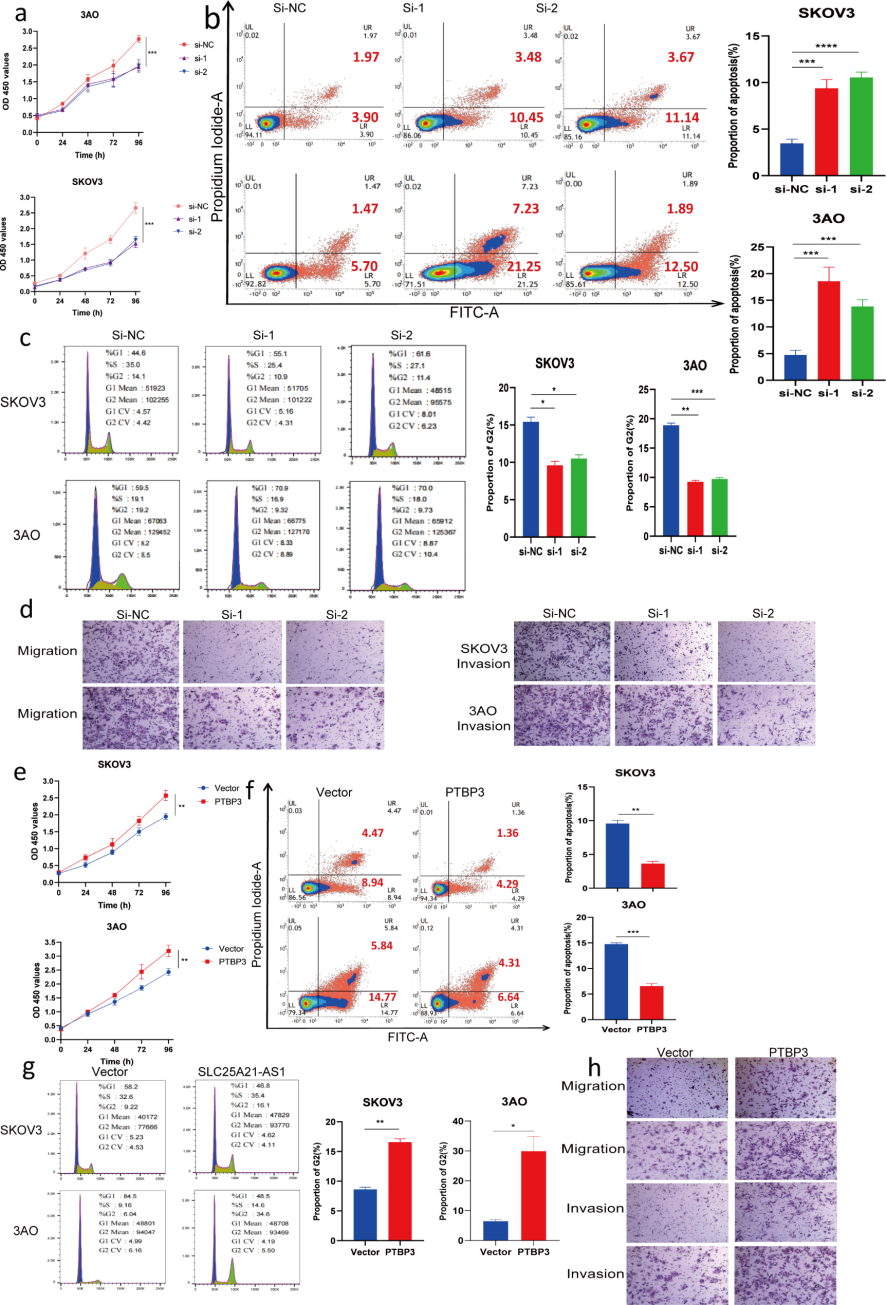
The function of ovarian cancer cells by regulating PTBP3. **a** After knocking down PTBP3, the proliferation rate of EOC cells was slowed down within 96 h. Data are mean ± SD from three independent replicate experiments. ***P<0.001. **b** Increased apoptosis of EOC cells after knockdown of PTBP3. Error bars represent the mean ± SD of three independent replicates. **c** The percentage of cells in G2 phase decreased after knockdown of PTBP3 (*n*=3). **d** The migration and invasion rates of ovarian cancer cells were decreased after knockdown of PTBP3 (*n*=3). **e** The proliferation of ovarian cancer cells was significantly accelerated after overexpression of PTBP3. **P<0.01. **f** Overexpression of PTBP3 significantly reduced apoptosis. Error bars represent the mean ± SD of three independent replicates. ***P<0.001, **P<0.01.**g** The percentage of EOC cells in G2 phase was significantly increased after overexpression of PTBP3 (*n*=3). **h** The migration and invasion of ovarian cancer cells were significantly increased after overexpression of PTBP3, data represent mean ± SD. **P<0.01

**Fig. 6:**
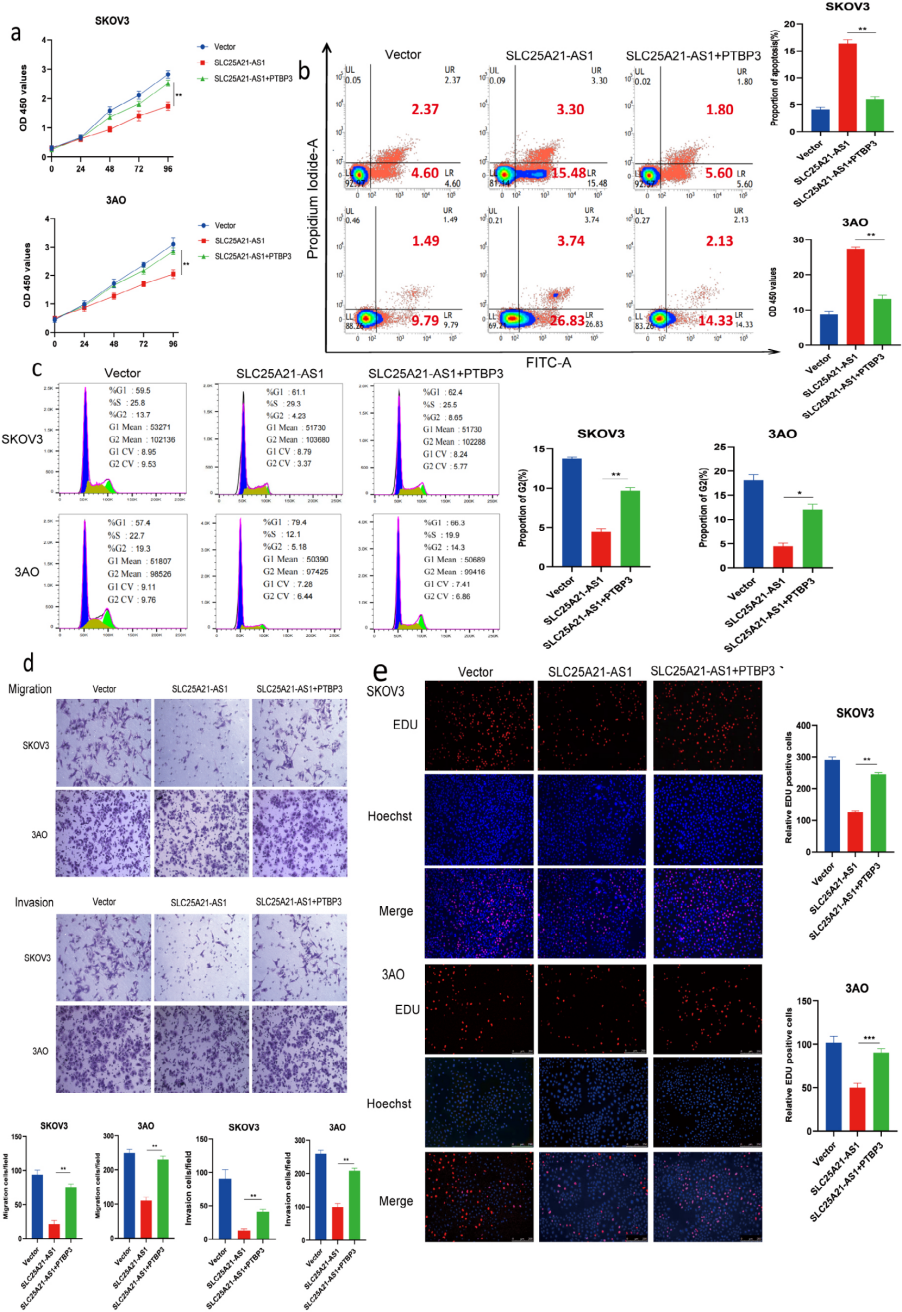
PTBP3 can reverse the inhibitory effect of SLC25A21-AS1 on EOC cells. **a** PTBP3 rescued the inhibitory effect of SLC25A21-AS1 on EOC cells proliferation (*n*=3). **b** Overexpression of both SLC25A21-AS1 and PTBP3 reduced the apoptosis of EOC cells compared with the SLC25A21-AS1 group**P<0.01. **c** The addition of PTBP3 with SLC25A21-AS1 re-increased the percentage of ovarian cancer cells in G2 phase. **d** PTBP3 effectively rescued the inhibitory migration and invasion effects of SLC25A21-AS1 (*n*=3). **e** EdU assay to detect the role of SLC25A21-AS1 and PTBP3 in ovarian cancer cell proliferation. Error Bars represent the mean ± SD of three independent replicate experiments. ***P<0.001, **P<0.01

## Electronic supplementary material

Below is the link to the electronic supplementary material.

Supplementary Material 1

