# Supplementary material for: Long non-coding RNA SLC25A21-AS1 inhibits the development of epithelial ovarian cancer by specifically inducing PTBP3 degradation
Source: Biomark Res. 2023 Jan 30;11:12. doi: 10.1186/s40364-022-00432-x (PMC9885650; doi:10.1186/s40364-022-00432-x)
Supplement: Supplementary file 1 — Additional file 1. [file 40364_2022_432_MOESM1_ESM.docx]

**Supplementary information**


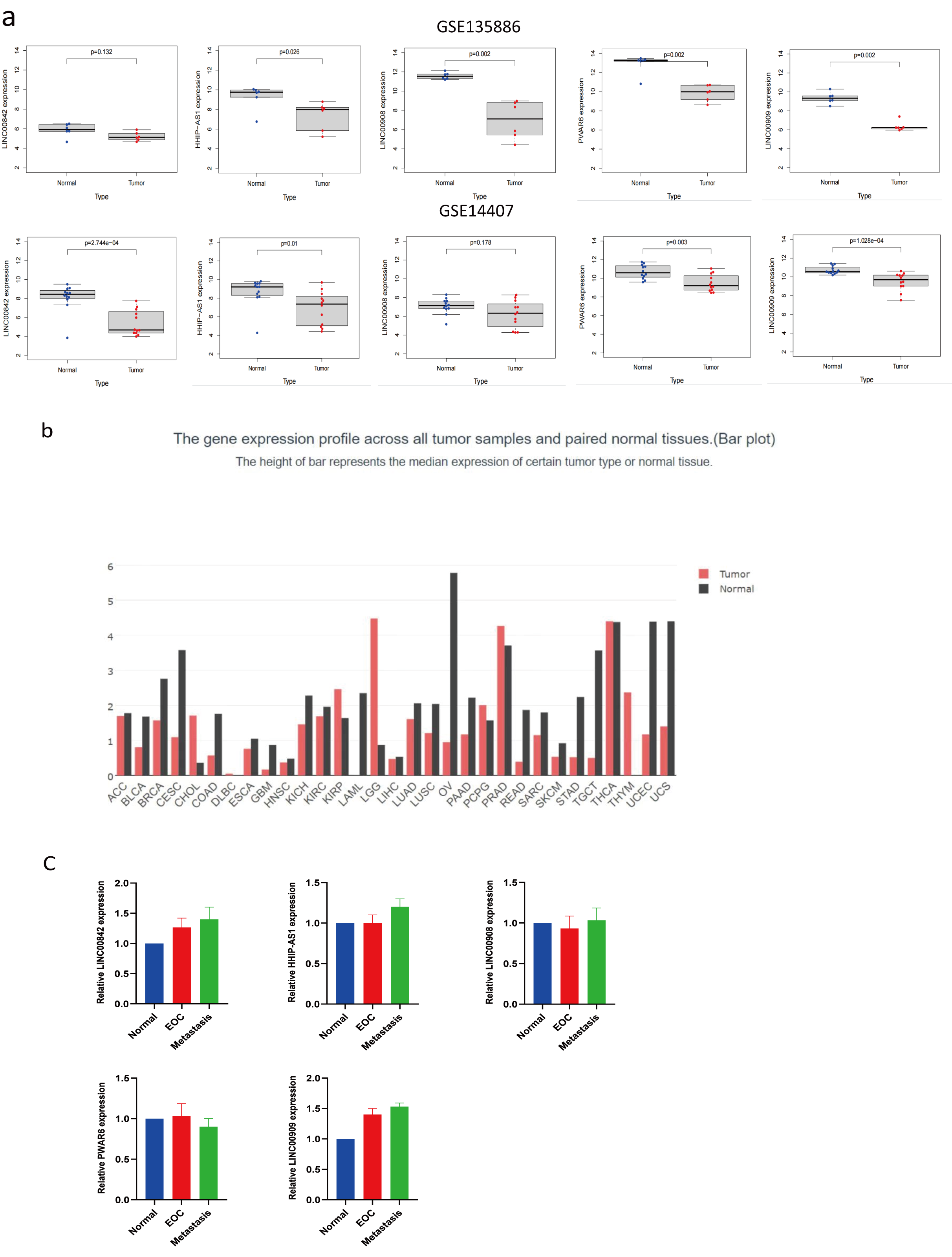


**S1.** **a.** The expression of LINC00842, HHIP-AS1, LINC00908, PWAR6 and LINC00909 in normal and tumors both in GSE135886 and GSE14407.The clinical data analysis was identified by package Limma using R software (version3.6.2). **b.**The gene expression profile of SLC25A21-AS1 across all tumor samples and paired normal tissues on Gene Expression Profiling Interactive(GEPIA). **c.** The expression of LINC00842, HHIP-AS1, LINC00908, PWAR6 and LINC00909 in EOC and the metastasis tissues.


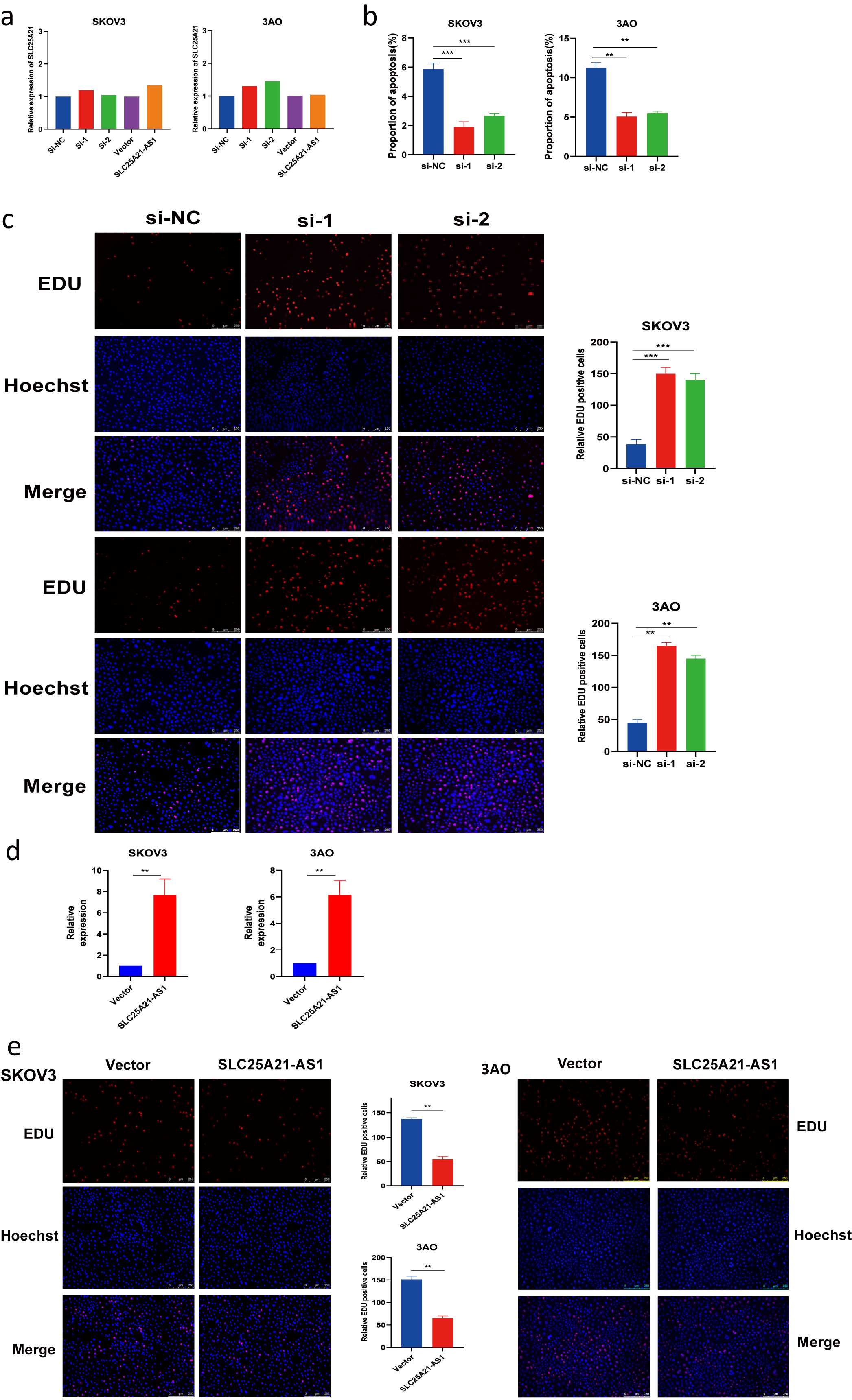


**S2.**Regulate the effect of SLC25A1-AS1 on SLC25A21 and the proliferation of EOC cells.**a.** Effects of knockdown or overexpression of SLC25A21-AS1 on maternal SLC25A21. **b.** Knockdown efficiency after knockdown of SLC25A21-AS1 by siRNA. Datas are from three independent replicate experiments and the mean ± SD. ***P<0.001, **P<0.01. **c.** EdU assay to detect the proliferation level of EOC cells after knockdown of SLC25A21-AS1. DAPI stands for nucleus and EdU stands for the number of cells in S phase (n=3). **d.** The overexpression efficiency of SLC25A21-AS1 after using the overexpression plasmid to transform into EOC cells. ***P<0.001. **e.** , the cell proliferation was detected by EdU assay after overexpression of SLC25A21-AS1. Datas are taken from the mean ± SD of replicate experiments.


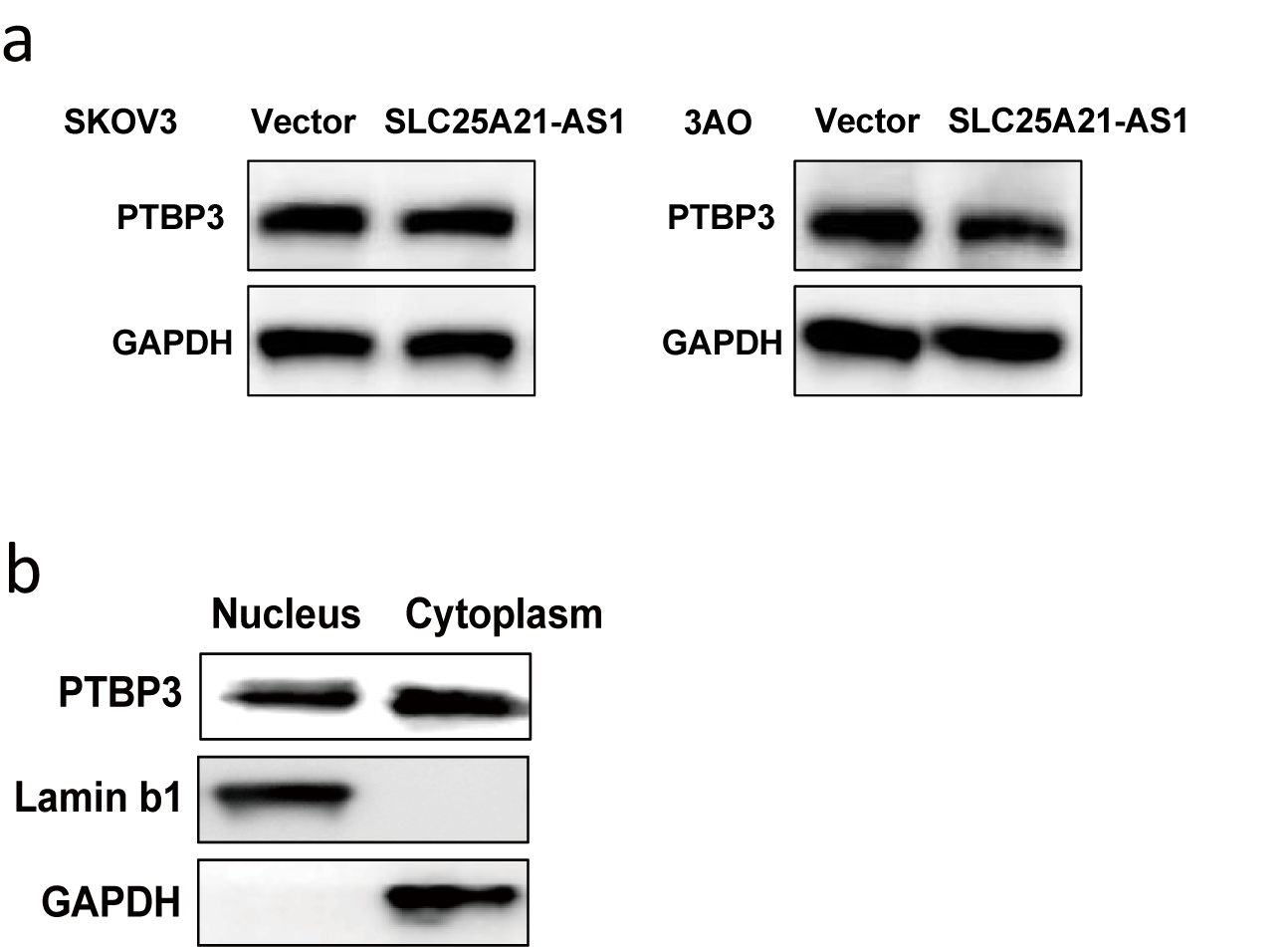


**S3.** The input of RIP and the detection of nucleocytoplasmic separationb. **a.** The input expressions of PTBP3 in RIP assays. **b.** The localization of PTBP3 in EOC cells was detected by nucleocytoplasmic separation assay.


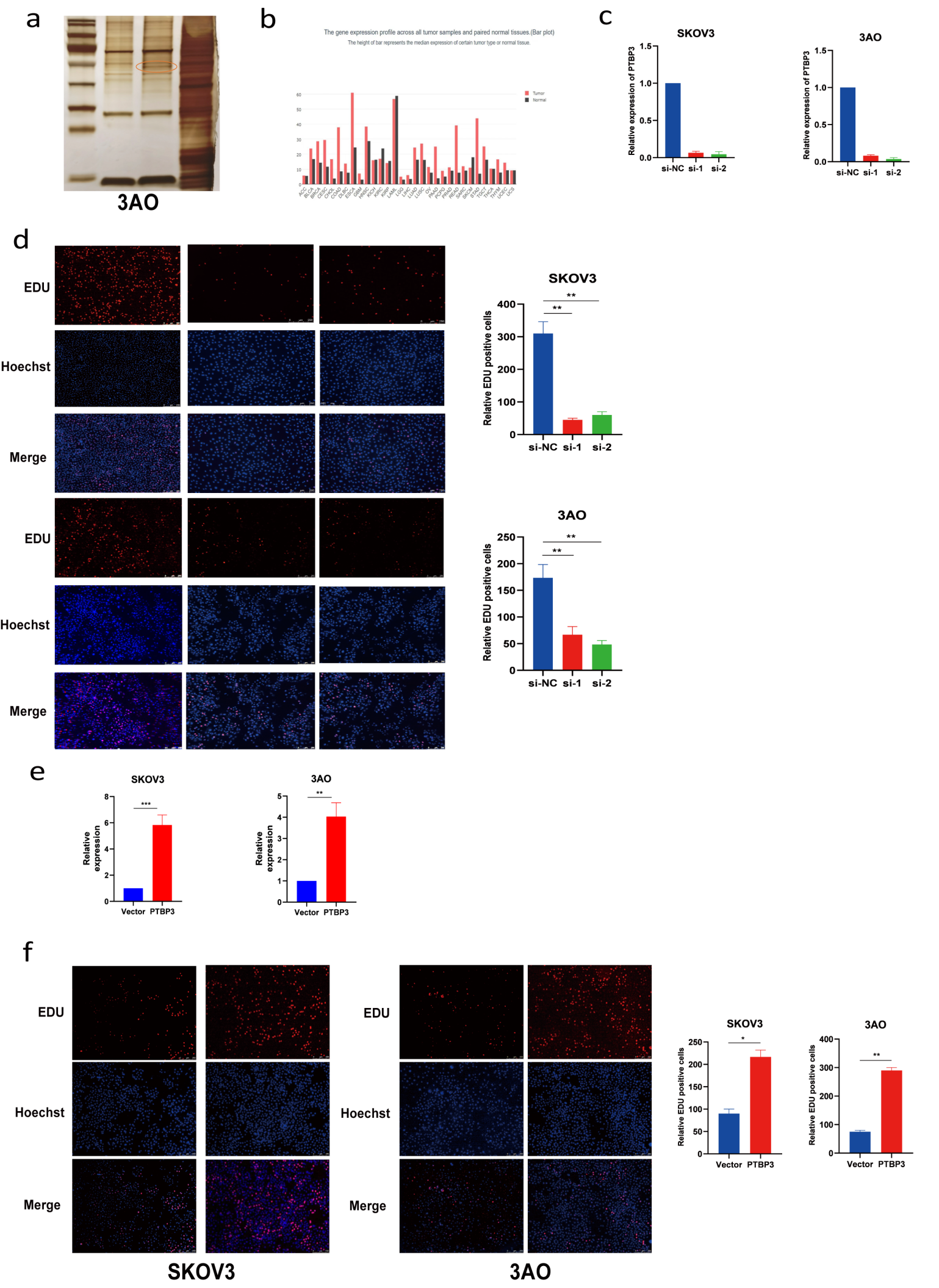


**S4.** Screening of PTBP3 and validation of its function in EOC cells. **a.** 3AO cells RNA pull-down assay and silver staining to find specific bands. Specific band is marked in orange circles. **b.** The gene expression profile of PTBP3 across all tumor samples and paired normal tissues on Gene Expression Profiling Interactive (GEPIA). **c.** Knockdown efficiency after knockdown of PTBP3 by siRNA (n=3). **d.** The proliferation level of EOC cells was detected by EdU assay after knockdown of PTBP3. Datas are mean ± SD from three independent replicate experiments. **e.** Overexpression efficiency after overexpression of PTBP3 using an overexpression plasmid. **f.** The effect of overexpression of PTBP3 on the proliferation of EOC cells. **P<0.01, **P<0.05.


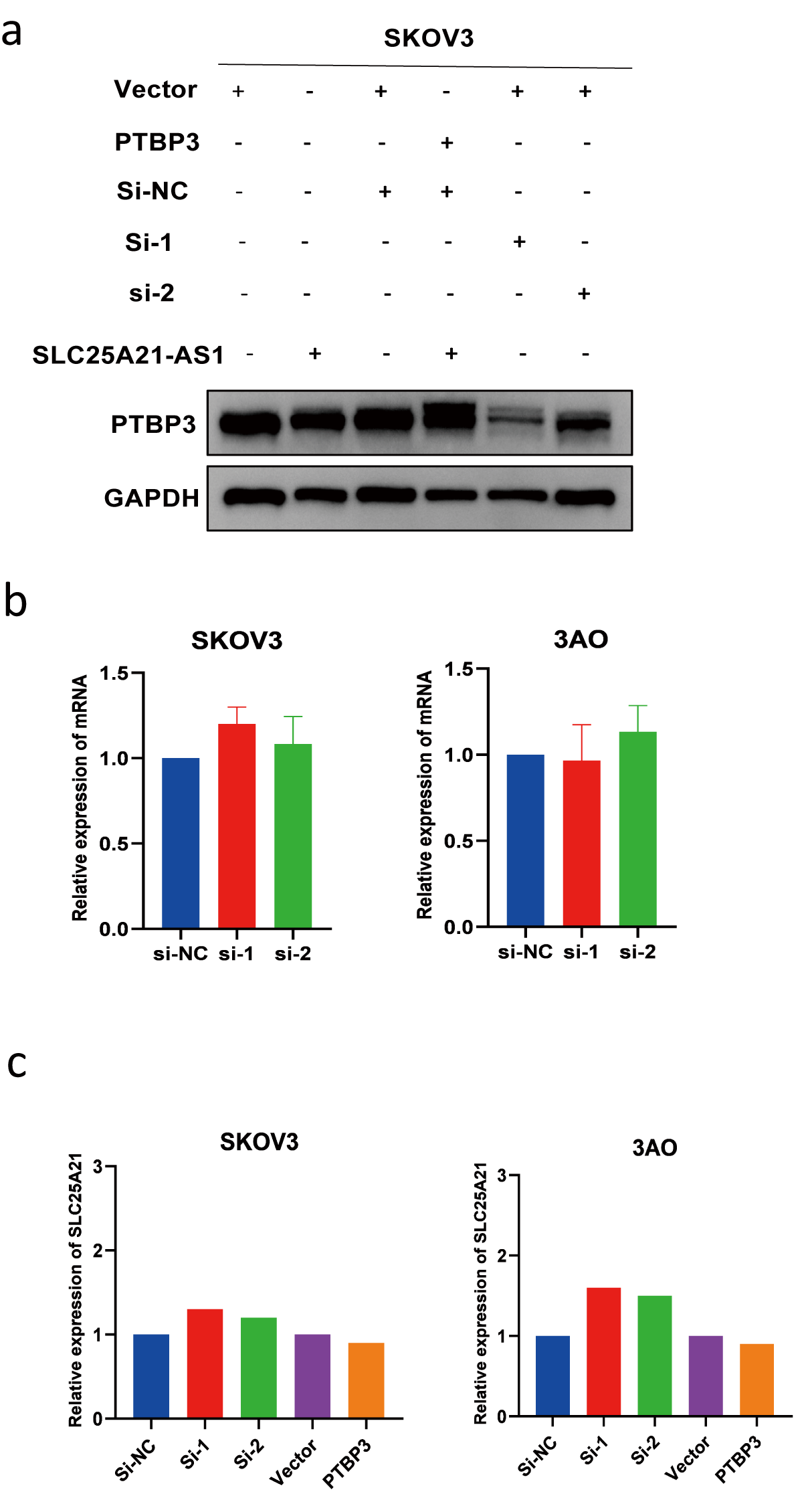


**S5.** The regulatory relationship between PTBP3 and SLC25A21-AS1 respectively and examined the effects on mRNA of PTBP3 and SLC25A21 expression.**a.** The effect of regulating SLC25A21-AS1 and PTBP3 on the expression of PTBP3.**b.** The effect of knockdown of SLC25A21-AS1 on PTBP3 mRNA. **c.** Real-time quantitative PCR was used to detect the effect of regulating PTBP3 on the maternal SLC25A21.


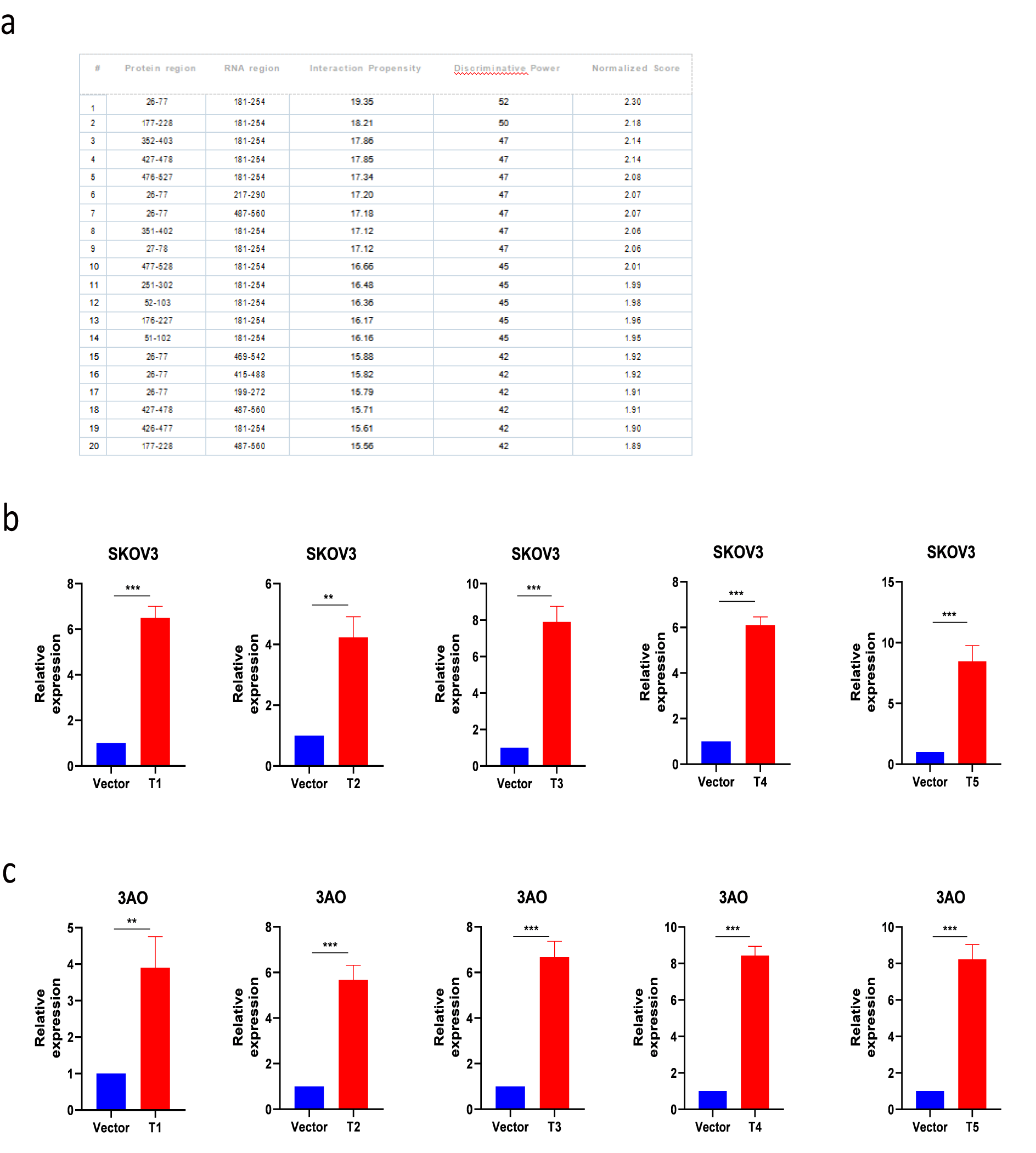


**S6.** Validation of the interaction site of PTBP3 and SLC25A21-AS1 and truncated body efficiency. **a.** Using catRAPID to predict the localization of PTBP3 interaction with SLC25A21-AS1. **b-c.** Overexpression efficiency after overexpressed T1-T5 in EOC cells using different truncated overexpression plasmids (n=3). ***P<0.001, **P<0.01.


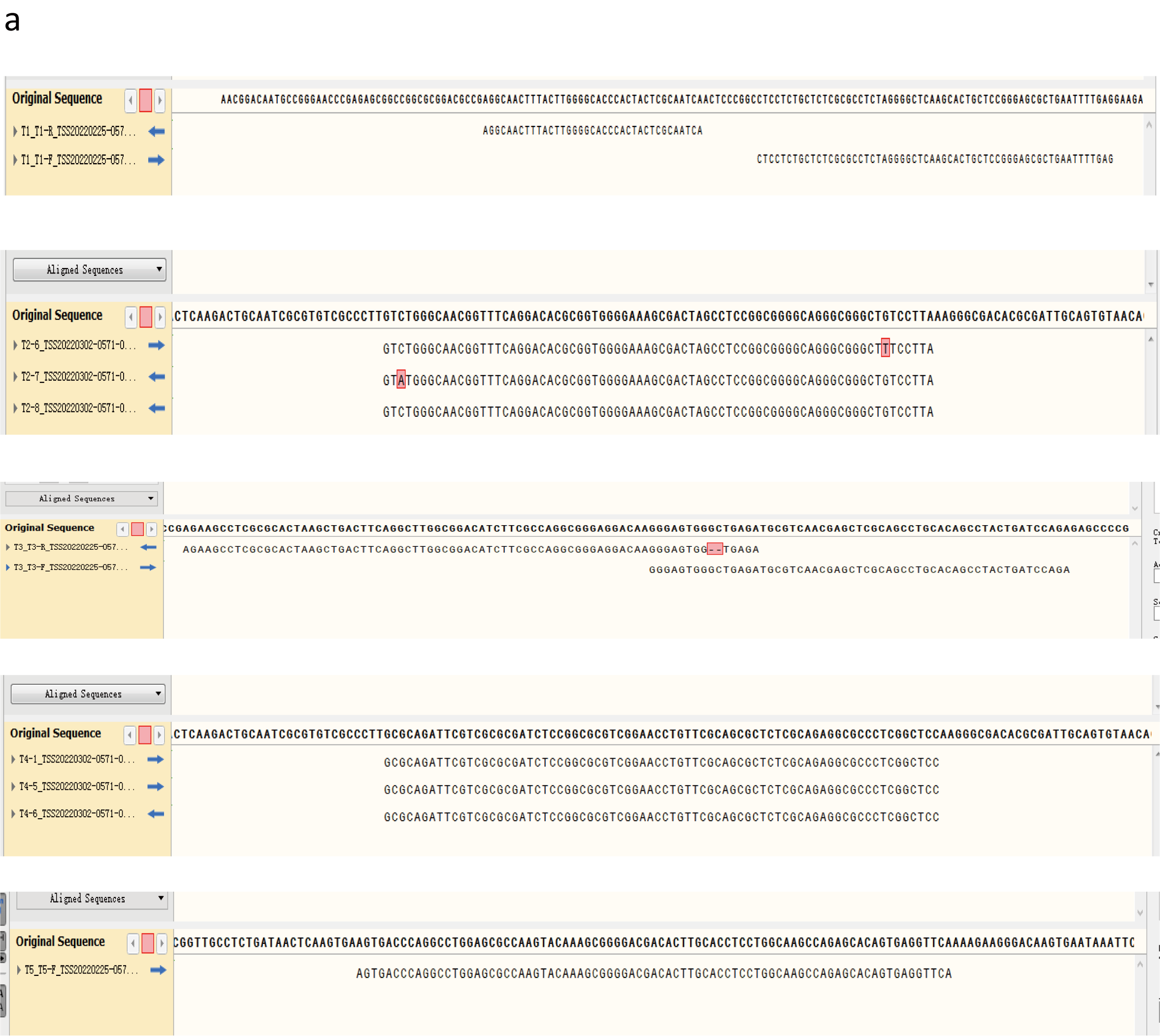


**S7. a.** Sequencing of the amplified products of different truncations, indicating that the products amplified by real-time quantitative PCR in the RIP assay are all corresponding truncations fragments.


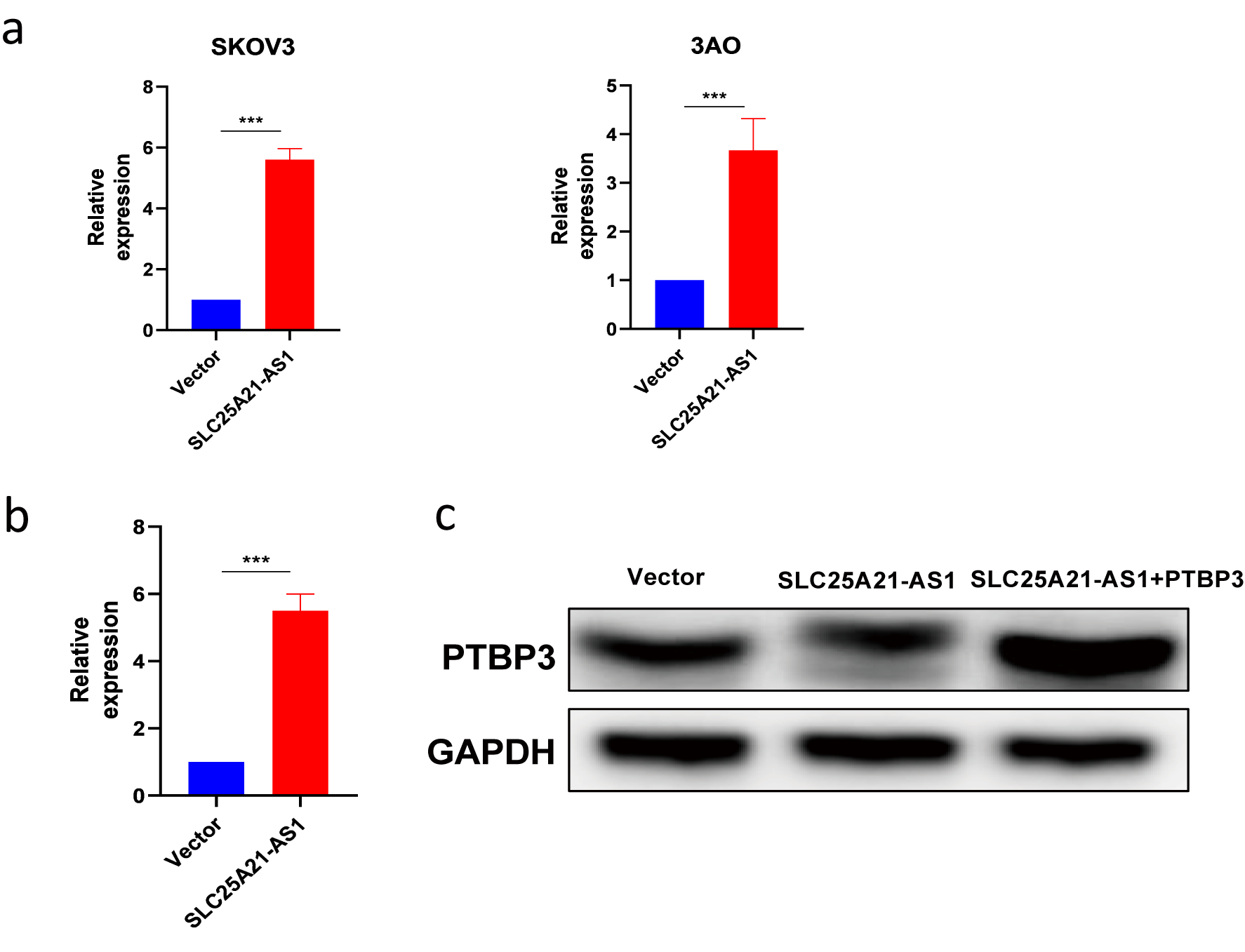


**S8.** SLC25AS21-AS1 and PTBP3 efficiency and expression verification of in vivo experiments. **a.** Overexpression efficiency of EOC cells stably expressing SLC25A21-AS1 (n=3). **b.** The overexpression efficiency of the SLC25A21-AS1 group was detected by real-time quantitative PCR (n=7) by removing a part of the tumor dissected from the in vivo experiments. **c.** PTBP3 expression in different groups in animal tumors.


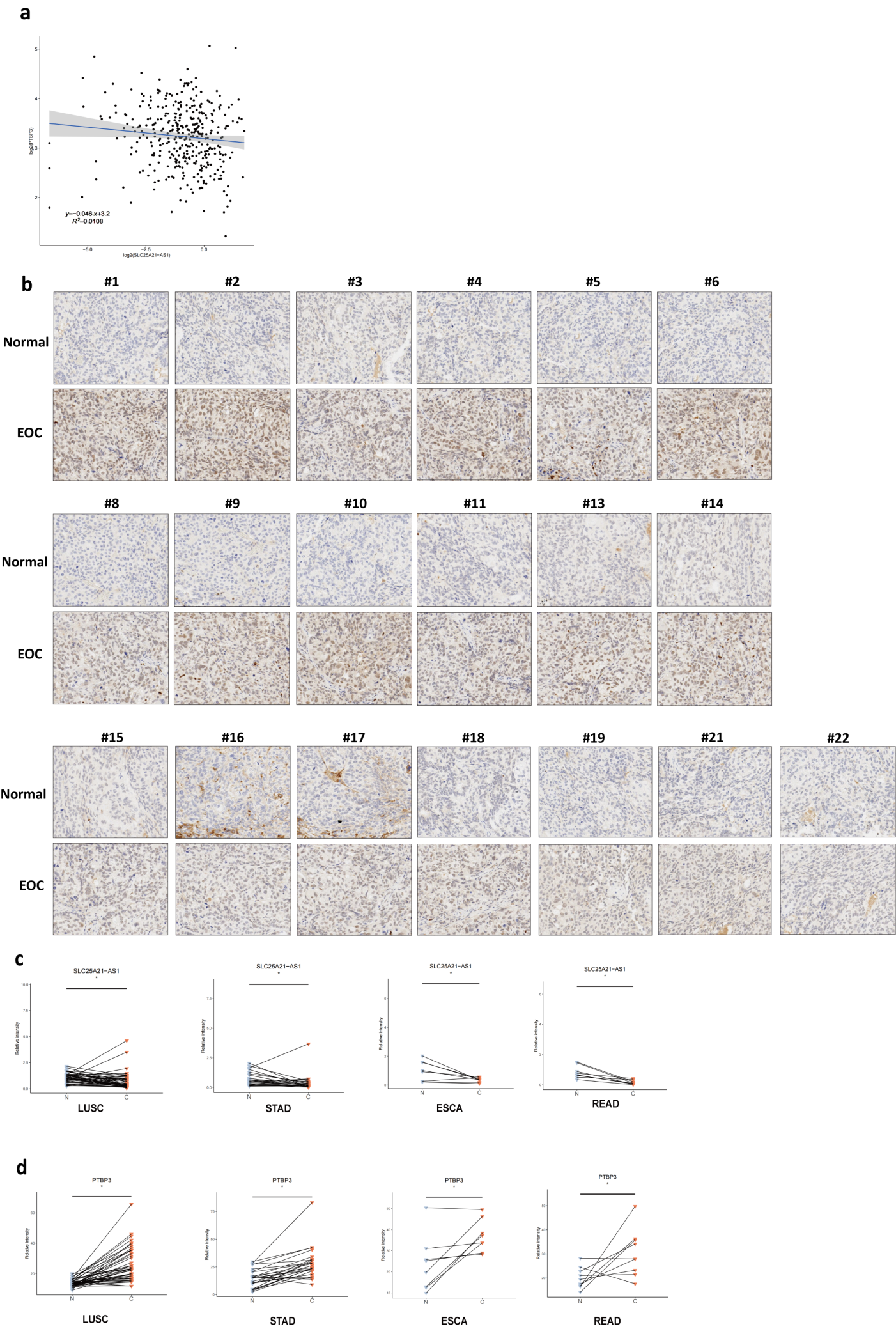


**S9.** Correlation analysis of PTBP3 and SLC25A21-AS1 and clinical significance in other tumors. **a.** The correlation analysis between SLC25A21-AS1 and mRNA of PTBP3, R^2^=0.0108 means there was almost no correlation. The gray area represented the 95% confidence interval. **b.** Immunohistochemical staining for PTBP3 protein expression in clinical tissue specimens. **c-d.** The relative expression differences of SLC25A21-AS1 and PTBP3 in other kind of tumor patients. Each line represents a patient.

**Table S1. Oligonucleotides sequences related to Figures 1-6 and Figures S2-8.**

The table includes siRNA sequences (Figures 2, 3 and 4; Figures S2, S3, S4 and S5), Biotin labelled probes (Figure 3,4; Figures S4), qRT-PCR and RT-PCR primers (Figures 1,2,3,4,5;Figures S2,S3,S4,S6,S8)

**Sequence (5’-3’)**

**siRNAs**

si-SLC25A21-AS1-1 CAGUUAGUUGUCCUAUAAUTT

si-SLC25A21-AS1-2 GUUAUGUAGGAGAGAUAUATT

si-PTBP3-1 CCAAUCACAGAGAACUUAATT

si-PTBP3-2 CCCUGUUACCCUGGAAGUUTT

**Primers used in qRT-PCR**

**Name**

**Forward**

**Reverse**

**Sequence (5’-3’)**

GAPDH TCACCACCATGGAGAAGGC GCTAAGCAGTTGGTGGTGCA

SLC25A21-AS1 TCGGCTCCGAAAATGTCTCT CTGATAGAGAAGGGCGTGGG

A2M-AS1 TGTGAGTGGCTTACCTTTGCT TCCCACAACAAACTCCTTCCT

USP12-AS2 CCCGCTGCCTAAACAGAAAAC CAGAGCAGGTTTCCTCCCAC

KANSL1-AS1 ACAGTCCCTCTTCCTCAGACA TGGCATCTTCAGGCTTGCTG

FAM66C ACACGAGTGCATGTGACCTT TCTGCTCCACTTGCCAACAT

ACVR2B-AS1 TCACCAGAGCAGCAGGAACAC GGATTATAGCAAGCAGGGACCAT

ZNF337-AS1 GTAGCCCTCTGGATTCTGTCTG GACCACAGGAGACTGGTGAGAAG

PTBP3 ATGGCGGATGCAAATCAAGC GATGCAGAGTGGCTGATGGT

T1 AGGCAACTTTACTTGGGGCA CTCAAAATTCAGCGCTCCCG

T2 GTAAGGAGACCTGTCTGGGCAA TGGTTCTGCAGGTAAGGACA

T3 CTCGCGCACTAAGCTGACTT GGATCAGTAGGCTGTGCAGG

T4 GCGCAGATTCGTCGCGCGAT GGAGCCGAGGGCGCCTCTG

T5 ATCAGGAGCCTCCGGTAGTT TGAACCTCACTGTGCTCTGG

SLC25A21 CAGAGATGTGCAACCGATCCA GGGGTTTCAGCCAAGATAGGT

**Biotin labelled probes**

**Sequence (5’-3’)/Details**

ChIRP Probe_1 ACTTGAGTTATCAGAGGCAA-/3bio/

ChIRP Probe_2 CTGATGCTGTGGGTACTAAA-/3bio/

ChIRP Probe_3 ATAGGACAACTAACTGAGGA-/3bio/

ChIRP NC_1 GGATCCATAGATATTGCGAA-/3bio/

ChIRP NC_2 TCGGAAGATTTGTGACGACT-/3bio/

ChIRP NC_3 ACGAATAAGAATGATCACGG-/3bio/

WT probe GTCTGGGCAACGGTTTCAGGACACGCGGTGGGGAAA

GCGACTAGCCTCCGGCGGGGCAGGGCGGGCTGTCCTTA

Mut probe

TGAGTTTACCATTGGGACTTCACATATTGTTTTCCCTATCAGCTAAGAATTATTTTACTTTATTTAGTGAAGGCTATCAGCTAAGAATTATTTTACTTTATTTAGTGAAGGC

**Details**

**Fish probe**

FISH-Probe Mix lnc1102084 RibioTM h-SLC25A21-AS1_ (Red,20T)

**Table S2. Protein profile (Top 20)**

**Mol. weight [kDa]**

**Unique peptides B**

**Unique peptides A**

**Score**

**Accession**

sp|O95758|PTBP3 77.331 0 8 59.689

sp|Q92945|KHSRP 49.598 0 7 49.598

sp|P27695|APE1 44.864 1 5 35.554

sp|P43034|LIS1 31.064 0 4 46.637

sp|Q8N6T3|ARFG1 26.24 0 4 44.667

sp|Q14320|FA50A 19.829 1 3 40.241

sp|Q15008|PSMD6 19.794 0 3 45.531

sp|P51398|RT29 19.093 0 2 45.566

sp|Q15758|AAAT 17.559 0 3 56.598

sp|P52294|IMA5 17.215 0 2 60.221

sp|Q5VW32|BROX 14.896 0 2 46.476

sp|P40222|TXLNA 12.424 0 2 61.89

sp|Q9H9P8|L2HDH 12.165 0 2 50.315

sp|Q9Y4P3|TBL2 11.994 0 1 49.797

sp|Q9Y570|PPME1 11.957 0 2 42.315

sp|P30153|2AAA 11.92 0 2 65.308

sp|Q9Y6G9|DC1L1 11.565 0 2 56.578

sp|Q9Y4X5|ARI1 11.075 0 2 64.117

sp|Q12849|GRSF1 10.954 0 2 53.126

sp|Q9BQ67|GRWD1 10.569 0 2 49.419

**Table S3. Subject details related to Figure 6F.**

**Stage**

**Sex**

**Age**

**ID**

EOC#1 Female 65 III

EOC#2 Female 57 II

EOC#3 Female 59 IV

EOC#4 Female 49 III

EOC#5 Female 47 II

EOC#6 Female 44 III

EOC#7 Female 43 III

EOC#8 Female 37 II

EOC#9 Female 58 III

EOC#10 Female 64 III

EOC#11 Female 68 IV

EOC#12 Female 65 III

EOC#13 Female 49 II

EOC#14 Female 58 III

EOC#15 Female 36 II

EOC#16 Female 42 III

EOC#17 Female 55 III

EOC#18 Female 41 III

EOC#19 Female 61 II

EOC#20 Female 40 III

EOC#21 Female 62 III

EOC#22 Female 65 III

EOC#23 Female 48 II

EOC#24 Female 50 III
